# Exploring of N-phthalimide-linked 1,2,3-triazole analogues with promising ­anti-SARS-CoV-2 activity: synthesis, biological screening, and molecular modelling studies

**DOI:** 10.1080/14756366.2024.2351861

**Published:** 2024-06-07

**Authors:** Ateyatallah Aljuhani, Mosa Alsehli, Mohamed A. Seleem, Shaya Y. Alraqa, Hany E. A. Ahmed, Nadjet Rezki, Mohamed R. Aouad

**Affiliations:** a Chemistry Department, College of Sciences, Taibah University, Saudi Arabia; b Pharmaceutical Organic Chemistry Department, Faculty of Pharmacy, Al-Azhar University, Nasr, City, Cairo, Egypt

**Keywords:** 123-Triazole, phtalamide, Schiff bases, COVID-19, molecular docking

## Abstract

In this study, a library of phthalimide Schiff base linked to 1,4-disubstituted-1,2,3-triazoles was designed, synthesised, and characterised by different spectral analyses. All analogues have been introduced for *in vitro* assay of their antiviral activity against COVID-19 virus using Vero cell as incubator with different concentrations. The data revealed most of these derivatives showed potent cellular anti-COVID-19 activity and prevent viral growth by more than 90% at two different concentrations with no or weak cytotoxic effect on Vero cells. Furthermore, *in vitro* assay was done against this enzyme for all analogues and the results showed two of them have IC50 data by 90 µM inhibitory activity. An extensive molecular docking simulation was run to analyse their antiviral mechanism that found the proper non-covalent interaction within the Mpro protease enzyme. Finally, we profiled two reversible inhibitors, COOH and F substituted analogues that might be promising drug candidates for further development have been discovered.

## Graphical abstract

**Figure:**
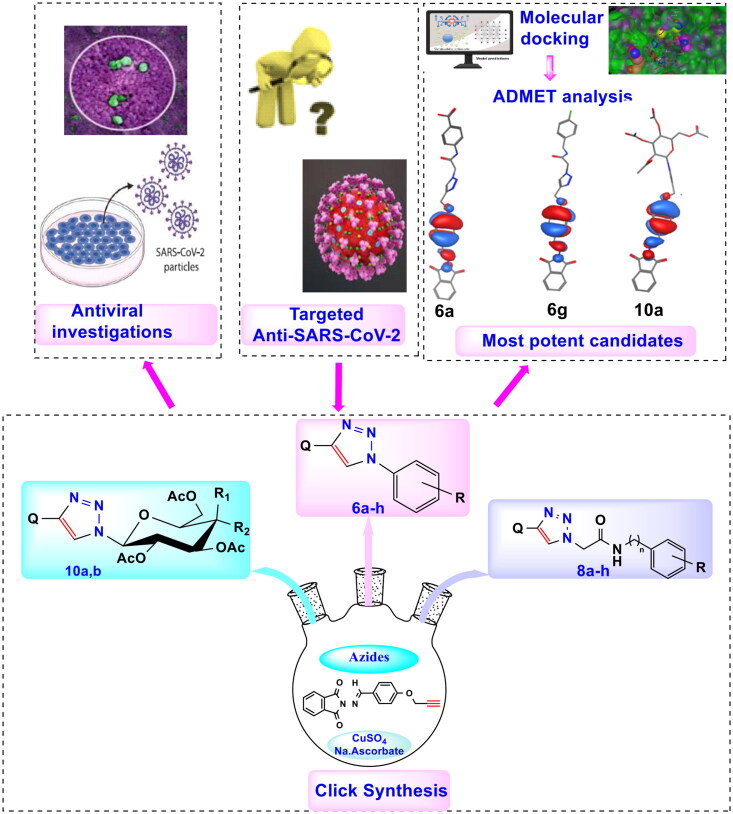

## Introduction

The ongoing COVID-19 pandemic has highlighted the urgent need for effective antiviral agents^1^. One promising class of compounds that has attracted considerable attention in this context is 1,2,3-triazole-carrying scaffolds^2^. These compounds possess diverse pharmacological activities and have demonstrated potential as antiviral agents. Their unique structural features, such as the ability to form hydrogen bonds and effectively interact with biological targets, make them an attractive scaffold for drug design and development^2^.

The rational design and synthesis of novel 1,2,3-triazole derivatives present a reasonable rationale for the discovery of new anti-COVID agents. By leveraging the structural versatility of the triazole moiety, researchers have been able to fine-tune the physicochemical and pharmacokinetic properties of these compounds, thereby enhancing their bioactivity and therapeutic potential^3–8^. The aims to explore the design, synthesis, and biological evaluation of 1,2,3-triazole derivatives with the specific goal of developing potent antiviral agents targeting COVID-19. Also, the work highlights the structure–activity relationships (SAR) of these derivatives, shedding light on the crucial structural motifs that confer potent antiviral effects.

**Figure 1.**
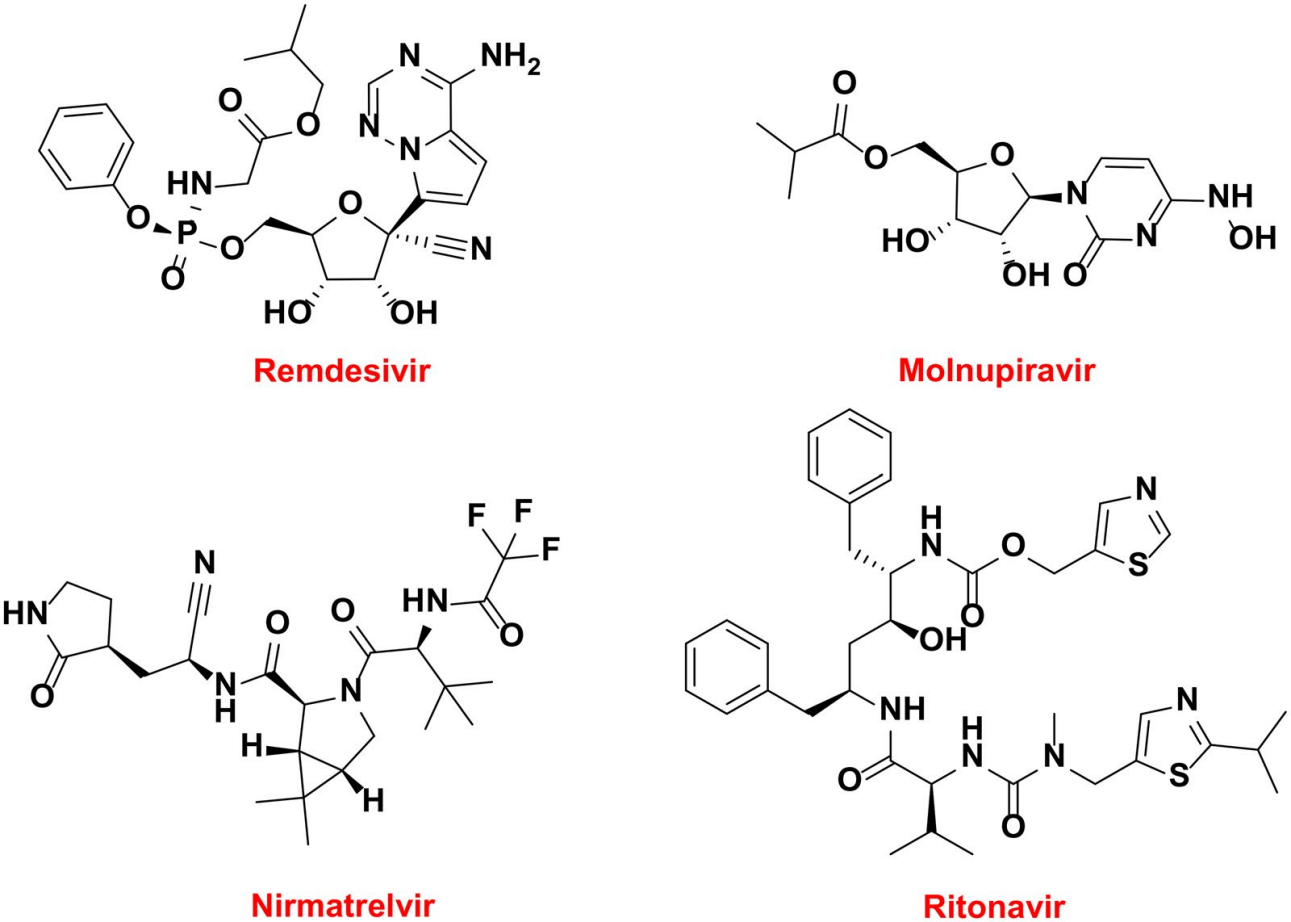
Used drugs for the treatment of SARS-CoV-2 infection.

Therefore, novel small molecule therapeutics exhibiting good oral bioavailability are urgently needed^9^. Results from two anti-SARS-CoV-2 oral clinical trials were recently published. In a phase-3 clinical trials, it was discovered that Moldupiravir (Figure 1), an inhibitor of viral RNA-dependent RNA polymerase (RdRp), reduced the risk of death or hospitalisation in adult patients with mild-to-moderate COVID-19 by 30% who were not hospitalized^10^. However, an 89% decrease in hospitalisation or mortality was observed when ritonavir and Nirmatrelvir (Figure 1) were used in conjunction as viral protease inhibitors^11^. These findings suggested that in order to prevent future pandemics, other mechanisms for oral anti-SARS-CoV-2 medications must be developed^12–14^.

Moreover, click chemistry-a low-cost method of catalysing the reaction between azides and alkynes – allows for the simple synthesis of 1,2,3-triazoles. Click chemistry is commonly employed not only to create derivatives of triazoles but also to fuse two or more chemical groups together to form a single hybrid molecule with enhanced biological activity^15–18^. The phthalimide group, being a derivative of triazoles, has demonstrated its utility in forming hybrid compounds that have antiviral action against HIV^19^, CMV, and varicella-zoster^20^. In addition, the importance of similar molecules was highlighted employing DFT theory for quantum computations and drug-like characters^21^ (ref 1 in the reviewer comments). Aromatic Schiff bases with azo linkage have also been emerged as an important scaffold in both medical and non-medical applications^8^^,^^22^(ref 2 in the reviewer comments). Crucially, compounds of phthalimide and triazole both exhibit significant anti-inflammatory efficacy^23^^,^^24^. As a result, 1,2,3-triazole-phthalimide hybrids may be able to treat the severe form of COVID-19, which is characterised by a powerful inflammatory process known as a "cytokine storm," in addition to preventing viral replication^13^^,^^25^^,^^26^.

Enzyme inhibition serves as a crucial strategy for curing several diseases including viral and bacterial infections. Several enzymes such as carbonic anhydrase and acetylcholinesterase disrupting key biological processes essential for treatment of wide range of diseases.^28^ Targeted inhibition of specific enzymes involved in vital pathways can impede the infectious agents, offering a promising avenue for the development of antiviral and antibacterial therapeutics. Similarly, SARS-CoV-2 enzyme inhibitors, especially M^pro^, the key protease, was proved as one of the fundamental strategies to impact this virus.^29^

Our study team forecast the design and synthesis of newer 1,2,3-triazole hybrid molecules within our research scope as continuation of our interest in this area and based on those reported in the literature^17^^,^^18^^,^^30–45^. Thus, we have anticipated on the synthesis of novel Schiff base tethering active phthalimide entity and lateral acetylenic side chain as an alkyne starting material for further cycloaddition with a series of organic azides, resulting in a novel array of 1,4-disubstituted-1,2,3-triazoles molecular hybrids.

### Work design

**Figure 2.**
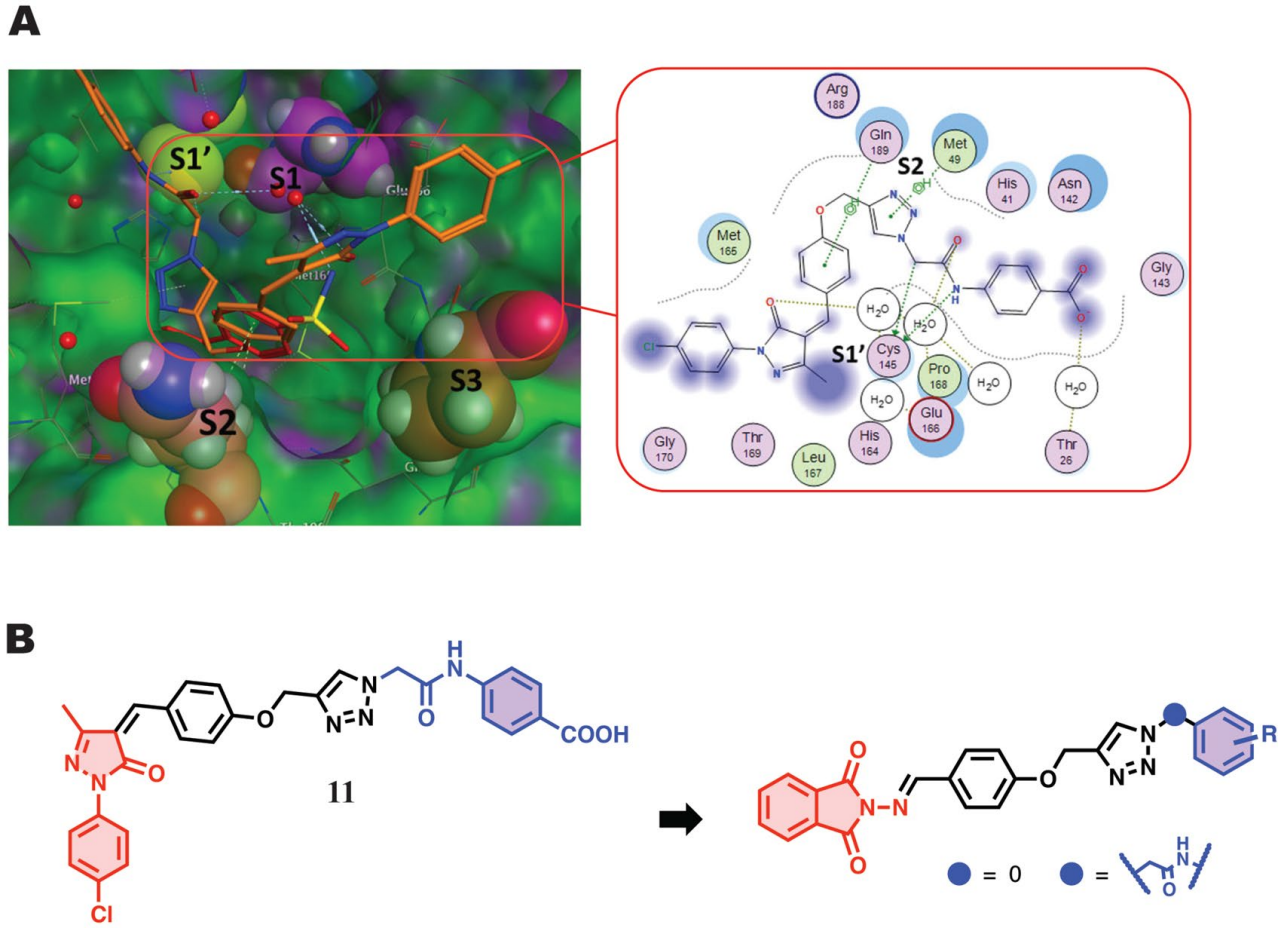
(A) docking pose of compound **11** in complex with M^pro^ (PBD ID 5R80) (left) and the 2D interaction map (right) showing the fundamental interactions of certain moieties in **11** with the protein; (B) The suggested modification aiming to improve its binding with the protein and enhance its anti-Covid activity.

Our previous investigation introduced efficient anti-SARS-CoV-2 analogues of molecular hybrids of rigid terminal scaffold bonded through heterocyclic basic triazole ring and ended with lipophilic-substituted fragments (Figure 2)^46^. Chemically, this compound and its congeners composed of phenylpyrazolone scaffold connected to lipophilic aryl moiety (4-acetamidobenzoic acid for active derivative) through 1,2,3-triazole linker. This compound inhibits SARS-CoV-2 growth in Vero 6 cells by 63.39 ± 0.48% at a concentration of 10 µM. In addition, a preliminary mechanistic study revealed that the scaffold linked to triazole entity could impacts SARS-CoV-2 by inhibition of the main protease M^pro^ machinery with IC_50_ 3.16 ± 1.2 µM with very low cytotoxic effect at higher concentrations^46^.

In addition, docking studies showed prominent binding to M^pro^ (PDB ID: 5R80) through two fundamental points. First, phenoxy and triazole parts interact with Gln189 and Met49 *via* hydrogen bonding (Figure 1(A)). These interactions highly stabilise the compound inside the binding pocket. Second, the amide linker of the lipophilic terminal part interacts with Cys145. On the other hand, the phenylpyrazole entity does not exhibit any meaningful prominent accommodation or binding to the active site residues^46^. Given the fundamental interactions exhibited by the middle region of this scaffold, we envisioned another round of chemical modification by the replacement of the phenylpyrazole part with chemical isosteric moiety bearing the same heteroatoms; *N*-aminophthalimide fragment^46^. Phthalimide has emerged as a privileged scaffold in the drug discovery process. The promising biological activities (including anti-SARS-CoV-2) of some reported phthalimide derivatives encouraged us to decide this replacement^3–5^^,^^47–50^. In addition, we will try various substituted phenyl groups at the right part connected to the triazole core directly or through the ordinary methyl amide linker. Moreover, a polar fragment of nucleosides will also be investigated and compared to non-polar ones.

## Materials and methods

### General chemistry

All reagents and solvents used were of the highest quality of analytical reagent grade and were used without further purification. Fine chemicals and solvents were purchased from BDH Chemicals Ltd. and Sigma-Aldrich. Melting points were measured on a Stuart Scientific SMP1 and are uncorrected. TLC was performed on UV fluorescent Silica gel Merck 60 F254 plates, and the spots were visualised using a UV lamp (254 nm). Fourier transform infra-red spectroscopy (FT-IR) was conducted on a Perkin-Elmer 1430 series FT-IR spectrometer. ^1^H and ^13^C nuclear magnetic resonance (NMR) spectra were obtained on a Bruker spectrometer (400 MHz) with TMS as an internal reference. Elemental analyses were performed using a GmbH-Vario EL III Elementar Analyser^51^.

### Synthesis and characterisation of 4-(prop-2-yn-1-yloxy) benzaldehyde (2)

Compound **2** was prepared in accordance with our previously published work^38^.

### Synthesis and characterisation of (E)-2-((4-(prop-2-yn-1-yloxy)benzylidene)amino)isoindoline-1,3-dione (4)

A stirring solution of compound **2** (5 mmol), 2-aminoisoindoline-1,3-dione (**3**) (5 mmol) and few drops of acetic acid in ethanol (40 ml) was heated under reflux for 4 h. After cooling, the resulting precipitate was collected by filtration and recrystallized from ethanol to give the desired Schiff base **4** as colourless crystals in 92% yield, mp: 183–184 °C. IR (υ, cm^−1^): IR (KBr): 1550 (C = C), 1700 (C = O, C = N), 2140 (C≡C), 3280 (≡CH).^1^H NMR (400 MHz, DMSO-d_6_): δ_H_ = 3.65 (*s*, 1H, ≡C**H**), 4.91 (*s*, 2H, OC**H**_2_), 7.15 (d, 2H, J = 8.0 Hz, Ar-**H**), 7.85–7.93 (*m*, 6H, Ar-**H**), 9.11 (*s*, 1H, **H**C = N). ^13^C NMR (100 MHz, DMSO-d_6_): δ_C_ = 54.09 (O**C**H_2_); 79.15 (C≡**C**H); 79.27 (**C**≡CH); 115.82, 123.86, 126.86, 130.35, 130.51, 135.34, 160.52, 165.02 (Ar-**C**, **C = N**, **C = O**). Calculated for: C_18_H_12_N_2_O_3_: C, 71.05; H, 3.97; N, 9.21. Found: C, 71.24; H, 3.90; N, 9.10.

### General click procedure for the synthesis of 1,4-disubstituted 1,2,3-triazoles 6a-h, 8a-h and 10a,b

A solution of O-propargylated Schiff Base **4** (1 mmol) in DMSO (10 ml) was added with stirring to a solution of copper sulphate (0.10 g) and sodium ascorbate (0.15 g) in water (10 ml). The reaction mixture was then stirred at 80 °C for 5–10 h with the appropriate phenylacetamide azides **5a-h**, aromatic azides **7a-h**, and/or glycosyl azides **9a,b** (1 mmol). TLC (hexane-ethylacetate) was used to monitor the reaction. After the reaction completed, the reaction mixture was poured onto crushed ice. The resulting precipitate was collected by filtration, washed with a saturated solution of ammonium chloride, then water, and recrystallized from ethanol/DMF to provide the desired 1,2,3-triazoles carrying phthalimide Schiff bases **6a-h**, **8a-h**, and/or **10a,b**.

### Characterisation of (E)-4–(2-(4-((4-(((1,3-dioxoisoindolin-2-yl)imino) methyl)phenoxy)methyl)-1H-1,2,3-triazol-1-yl)acetamido)benzoic acid (6a)

This compound was obtained as white pellets in 87% yield, mp: 264–265 °C. IR (υ, cm^−1^): 1575 (C = C), 1700 (C = N), 1730 (C = O), 2970 (CH-Al), 3045 (CH-Ar). ^1^H NMR (400 MHz, DMSO-d_6_): δ_H_ = 5.27 (*s*, 0.3H, OC**H_2_**), 5.31 (*s*, 1.7H, OC**H_2_**), 5.35 (*s*, 2H, NC**H_2_**CO), 7.12 (dd, 1H, *J* = 4 Hz, *J* = 8 Hz, Ar-**H**), 7.24–7.32 (*m*, 4H, Ar-**H**), 7.39–7.44 (*m*, 2H, Ar-**H**), 7.71–7.85 (*m*, 5H, Ar-**H**), 8.21 (*s*, 1H, C**H**-1,2,3-triazole), 9.08 (*s*, 0.3H, **H**C = N), 9.80 (*s*, 0.7H, **H**C = N), 10.12 (*s*, 1H, N**H**), 10.12 (*s*, 1H, COO**H**). ^13^C NMR (100 MHz, DMSO-d_6_): δ_C_ = 52.62 (N**C**H_2_); 61.70 (O**C**H_2_); 115.71, 115.80, 121.47, 123.70, 126.52, 130.37, 130.57, 135.24, 135.40, 142.32, 142.52, 159.90, 160.68, 160.78, 161.49, 164.59, 165.00 (Ar-**C**, **C = N**, **C = O**, **CO**OH). Calculated for: C_27_H_20_N_6_O_6_: C, 61.83; H, 3.84; N, 16.02. Found: C, 61.93; H, 3.89; N, 16.09.

### Characterisation of (E)-2–(4-((4-(((1,3-dioxoisoindolin-2-yl)imino) methyl)phenoxy)methyl)-1H-1,2,3-triazol-1-yl)-N-(2-iodophenyl) acetamide (6b)

This compound was obtained as white pellets in 88% yield, mp: 213–214 °C. IR (υ, cm^−1^): 1560 (C = C), 1690 (C = N), 1720 (C = O), 2930 (CH-Al), 3030 (CH-Ar). ^1^H NMR (400 MHz, DMSO-d_6_): δ_H_ = 5.23 (s, 0.3H, OC**H_2_**), 5.25 (s, 1.7H, OC**H_2_**), 5.36 (s, 2H, NC**H_2_**CO), 6.98 (dd, 1H, *J* = 4 Hz, *J* = 8 Hz, Ar-**H**), 7.15–7.21 (*m*, 3H, Ar-**H**), 7.33–7.40 (*m*, 3H, Ar-**H**), 7.79–7.88 (*m*, 5H, Ar-**H**), 8.25 (*s*, 1H, C**H**-1,2,3-triazole), 9.04 (*s*, 0.3H, **H**C = N), 9.82 (*s*, 0.7H, **H**C = N), 9.97 (*s*, 1H, N**H**). ^13^C NMR (100 MHz, DMSO-d_6_): δ_C_ = 52.46 (N**C**H_2_); 61.82 (O**C**H_2_); 96.65, 115.71, 115.78, 123.91, 127.17, 127.77, 128.71, 129.35, 130.33, 130.52, 132.36, 139.12, 139.61, 160.95, 163.47, 165.08, 165.12 (Ar-**C**, **C = N**, **C = O**). Calculated for: C_26_H_19_IN_6_O_4_: C, 51.50; H, 3.19; N, 13.86. Found: C, 51.69; H, 3.27; N, 13.74.

### Characterisation of (E)-2–(4-((4-(((1,3-dioxoisoindolin-2-yl)imino) methyl)phenoxy)methyl)-1H-1,2,3-triazol-1-yl)-N-(2-fluoro-4-iodophenyl)acetamide (6c)

This compound was obtained as white pellets in 88% yield, mp: 228–229 °C. IR (υ, cm^−1^): 1580 (C = C), 1685 (C = N), 1725 (C = O), 2920 (CH-Al), 3090 (CH-Ar). ^1^H NMR (400 MHz, DMSO-d_6_): δ_H_ = 5.29, 5.32 (2s, 2H, OC**H_2_**), 5.42 (*s*, 2H, NC**H_2_**CO), 7.02 (*s*, 2H, J = 8 Hz, Ar-**H**), 7.25–7.45 (*m*, 5H, Ar-**H**), 7.85–7.90 (*m*, 5H, Ar-**H**), 8.31 (*s*, 1H, C**H**-1,2,3-triazole), 9.12 (*s*, 0.2H, **H**C = N), 9.88 (*s*, 0.8H, **H**C = N), 10.01 (*s*, 1H, N**H**). ^13^C NMR (100 MHz, DMSO-d_6_): δ_C_ = 52.46 (N**C**H_2_); 61.88 (O**C**H_2_); 96.49, 115.64, 115.76, 120.89, 123.75, 123.98, 126.52, 127.16, 127.66, 129.31,129.37, 130.35, 130.58, 132.19, 132.32, 135.42, 139.15, 139.64, 142.37, 163.64, 165.07 (Ar-**C**, **C = N**, **C = O**). Calculated for: C_26_H_18_FIN_6_O_4_: C, 50.02; H, 2.91; N, 13.46. Found: C, 50.19; H, 2.98; N, 13.57.

### Characterisation of (E)-2–(4-((4-(((1,3-dioxoisoindolin-2-yl)imino)methyl)phenoxy)methyl)-1H-1,2,3-triazol-1-yl)-N-(4-nitrophenyl)acetamide (6d)

This compound was obtained as white pellets in 90% yield, mp: 242–243 °C. IR (υ, cm^−1^): 1590 (C = C), 1675 (C = N), 1725 (C = O), 2920 (CH-Al), 3035 (CH-Ar). ^1^H NMR (400 MHz, DMSO-d_6_): δ_H_ = 5.30 (*s*, 2H, OC**H_2_**), 5.47 (*s*, 2H, NC**H_2_**CO), 7.24 (d, 2H, *J* = 8 Hz, Ar-**H**), 7.85–7.92 (*m*, 8H, Ar-**H**), 8.27 (d, 2H, *J* = 8 Hz, Ar-**H**), 8.33 (*s*, 1H, C**H**-1,2,3-triazole), 9.12 (*s*, 1H, **H**C = N), 11.12 (*s*, 1H, N**H**). ^13^C NMR (100 MHz, DMSO-d_6_): δ_C_ = 52.85 (N**C**H_2_); 61.71 (O**C**H_2_); 115.63, 115.78, 123.71, 124.00, 125.76, 126.53, 130.35, 142.71, 142.52, 143.09, 144.98, 159.90, 160.77, 161.48, 165.01, 165.81 (Ar-**C**, **C = N**, **C = O**). Calculated for: C_26_H_19_N_7_O_6_: C, 59.43; H, 3.64; N, 18.66. Found: C, 59.59; H, 3.57; N, 18.57.

### Characterisation of (E)-2–(4-((4-(((1,3-dioxoisoindolin-2-yl)imino) methyl)phenoxy)methyl)-1H-1,2,3-triazol-1-yl)-N-(4-fluorophenyl) acetamide (6e)

This compound was obtained as white pellets in 89% yield, mp: 219–220 °C. IR (υ, cm^−1^): 1590 (C = C), 1675 (C = N), 1720 (C = O), 2920 (CH-Al), 3035 (CH-Ar). ^1^H NMR (400 MHz, DMSO-d_6_): δ_H_ = 5.23 (*s*, 1.7H, OC**H_2_**), 5.26 (*s*, 0.3H, OC**H_2_**), 5.30 (*s*, 2H, NC**H_2_**CO), 7.11–7.21 (*m*, 4H, Ar-**H**), 7.52–7.56 (*m*, 2H, Ar-**H**), 7.76–7.88 (*m*, 6H, Ar-**H**), 8.25 (*s*, 1H, C**H**-1,2,3-triazole), 9.04 (*s*, 0.7H, **H**C = N), 9.82 (*s*, 0.3H, **H**C = N), 10.53 (*s*, 1H, N**H**). ^13^C NMR (100 MHz, DMSO-d_6_): δ_C_ = 52.63 (N**C**H_2_); 61.67 (O**C**H_2_); 115.71, 115.78, 115.98, 116.16, 121.57, 121.63, 123.38, 123.91, 126.52, 127.08, 130.52, 130.57, 132.37, 134.91, 135.24, 135.40, 157.83, 159.74, 160.94, 161.51, 164.63, 165.09 (Ar-**C**, **C = N**, **C = O**). Calculated for: C_26_H_19_FN_6_O_4_: C, 62.65; H, 3.84; N, 16.86. Found: C, 62.41; H, 3.91; N, 16.75.

### Characterisation of (E)-2–(4-((4-(((1,3-dioxoisoindolin-2-yl)imino) methyl)phenoxy)methyl)-1H-1,2,3-triazol-1-yl)-N-(4-methylbenzyl) acetamide (6f)

This compound was obtained as colourless crystals in 88% yield, mp: 201–202 °C. IR (υ, cm^−1^): 1560 (C = C), 1670 (C = N), 1715 (C = O), 2930 (CH-Al), 3070 (CH-Ar). ^1^H NMR (400 MHz, DMSO-d_6_): δ_H_ = 2.28 (*s*, 3H, C**H_3_**), 4.30 (d, 2H, *J* = 8.0 Hz, NHC**H_2_**), 5.23 (*s*, 2H, OC**H_2_**), 5.30 (*s*, 2H, NC**H_2_**CO), 7.20–7.32 (*m*, 5H, Ar-**H**), 7.39–7.48 (*m*, 5H, Ar-**H**), 7.81 (d, 2H, *J* = 8.0 Hz, Ar-**H**), 8.29 (*s*, 1H, C**H**-1,2,3-triazole), 9.05 (*t*, 1H, *J* = 8.0 Hz, N**H**), 9.80 (*s*, 1H, **H**C = N). ^13^C NMR (100 MHz, DMSO-d_6_): δ_C_ = 25.63 (**C**H_3_); 42.23 (NH**C**H_2_); 52.15 (N**C**H_2_); 61.70 (O**C**H_2_); 115.72, 125.32, 127.18, 128.70, 128.98, 129.45, 130.56, 131.16, 132.45, 137.45, 142.44, 163.23, 166.02 (Ar-**C**, **C = N**, **C = O**). Calculated for: C_28_H_24_N_6_O_4_: C, 66.13; H, 4.76; N, 16.53. Found: C, 66.31; H, 4.68; N, 16.64.

### Characterisation of (E)-2–(4-((4-(((1,3-dioxoisoindolin-2-yl)imino) methyl)phenoxy)methyl)-1H-1,2,3-triazol-1-yl)-N-(4-fluorobenzyl) acetamide (6 g)

This compound was obtained as colourless crystals in 90% yield, mp: 195–196 °C. IR (υ, cm^−1^): 1585 (C = C), 1690 (C = N), 1715 (C = O), 2920 (CH-Al), 3070 (CH-Ar). ^1^H NMR (400 MHz, DMSO-d_6_): δ_H_ = 4.33 (d, 2H, *J* = 8.0 Hz, NHC**H_2_**), 5.24 (*s*, 2H, OC**H_2_**), 5.29 (*s*, 2H, NC**H_2_**CO), 7.26–7.36 (*m*, 6H, Ar-**H**), 7.45–7.52 (*m*, 4H, Ar-**H**), 7.87 (d, 2H, J = 8.0 Hz, Ar-**H**), 8.32 (*s*, 1H, C**H**-1,2,3-triazole), 9.10 (*t*, 1H, J = 8.0 Hz, N**H**), 9.81 (*s*, 1H, **H**C = N). ^13^C NMR (100 MHz, DMSO-d_6_): δ_C_ = 42.36 (NH**C**H_2_); 52.11 (N**C**H_2_); 61.79 (O**C**H_2_); 115.69, 115.93, 127.45, 129.31, 130.25, 130.46, 131.84, 132.33, 139.46, 143.52, 157.52, 161.35, 163.56, 166.21 (Ar-**C**, **C = N**, **C = O**). Calculated for: C_27_H_21_FN_6_O_4_: C, 63.28; H, 4.13; N, 16.40. Found: C, 63.01; H, 4.02; N, 16.57.

### Characterisation of (E)-N-(4-chlorobenzyl)-2–(4-((4-(((1,3-dioxoisoindolin-2-yl)imino)methyl)phenoxy)methyl)-1H-1,2,3-triazol-1-yl)acetamide (6h)

This compound was obtained as colourless crystals in 90% yield, mp: 208–209 °C. IR (υ, cm^−1^): 1570 (C = C), 1680 (C = N), 1720 (C = O), 2940 (CH-Al), 3055 (CH-Ar). ^1^H NMR (400 MHz, DMSO-d_6_): δ_H_ = 4.31 (d, 2H, *J* = 8.0 Hz, NHC**H_2_**), 5.21 (*s*, 2H, OC**H_2_**), 5.28 (*s*, 2H, NC**H_2_**CO), 7.23–7.38 (*m*, 10H, Ar-**H**), 7.89 (d, 2H, J = 8.0 Hz, Ar-**H**), 8.26 (*s*, 1H, C**H**-1,2,3-triazole), 9.03 (*t*, 1H, *J* = 8.0 Hz, N**H**), 9.86 (*s*, 1H, **H**C = N). ^13^C NMR (100 MHz, DMSO-d_6_): δ_C_ = 42.12 (NH**C**H_2_); 52.05 (N**C**H_2_); 61.75 (O**C**H_2_); 115.63, 127.03, 128.75, 129.67, 130.25, 131.99, 132.33, 138.24, 142.23, 163.41, 166.05 (Ar-**C**, **C = N**, **C = O**). Calculated for: C_27_H_21_ClN_6_O_4_: C, 61.31; H, 4.00; N, 15.89. Found: C, 61.63; H, 4.10; N, 15.70.

### Characterisation of (E)-2-((4-((1–(4-fluorophenyl)-1H-1,2,3-triazol-4-yl)methoxy)benzylidene)amino) isoindoline-1,3-dione (8a)

This compound was obtained as colourless crystals in 87% yield, mp: 238–239 °C. IR (υ, cm^−1^): 1540 (C = C), 1670 (C = N), 1720 (C = O), 2930 (CH-Al), 3075 (CH-Ar). ^1^H NMR (400 MHz, DMSO-d_6_) δ_H_ = 5.37, 5.39 (2s, 2H, OC**H_2_**), 7.24–7.61 (*m*, 6H, Ar-**H**), 7.87–7.91 (*m*, 6H, Ar-**H**), 8.81 (*s*, 1H, C**H**-1,2,3-triazole), 9.12 (*s*, 0.7H, **H**C = N), 9.89 (*s*, 0.3H, **H**C = N). ^13^C NMR (100 MHz, DMSO-d_6_): δ_C_ = 61.51 (O**C**H_2_); 115.72, 117.51, 117.71, 123.91, 125.10, 126.07, 126.68, 126.87, 130.57, 131.92, 143.41, 153.10, 155.59, 160.68, 163.35, 164.99 (Ar-**C**, **C = N**, **C = O**). Calculated for: C_24_H_16_FN_5_O_3_: C, 65.30; H, 3.65; N, 15.87. Found: C, 65.50; H, 3.76; N, 15.75.

### Characterisation of (E)-4–(4-((4-(((1,3-dioxoisoindolin-2-yl)imino) methyl)phenoxy)methyl)-1H-1,2,3-triazol-1-yl)benzoic acid (8b)

This compound was obtained as pale yellow solid in 86% yield, mp: 287–288 °C. IR (υ, cm^−1^): 1570 (C = C), 1660 (C = N), 1710 (C = O), 2900 (CH-Al), 3060 (CH-Ar). ^1^H NMR (400 MHz, DMSO-d_6_) δ_H_ = 5.30 (*s*, 1.8H, OC**H_2_**), 5.33 (*s*, 0.2H, OC**H_2_**), 7.19 (d, 2H, J = 8 Hz, Ar-**H**), 7.81–7.86 (*m*, 9H, Ar-**H**), 8.23 (d, 1H, J = 4 Hz, Ar-**H**), 9.01 (*s*, 1H, C**H**-1,2,3-triazole), 9.05 (*s*, 0.7H, **H**C = N), 9.83 (*s*, 0.3H, **H**C = N), 12.15 (*s*, 1H, COO**H**). ^13^C NMR (100 MHz, DMSO-d_6_): δ_C_ = 61.64 (O**C**H_2_); 115.77, 115.84, 123.74, 123.91, 126.71, 130.54, 130.58, 132.38, 135.40, 139.94, 143.65, 160.84, 161.37, 165.07, 173.11 (Ar-**C**, **C = N**, **C = O**, **CO**OH). Calculated for: C_25_H_17_N_5_O_5_: C, 64.24; H, 3.67; N, 14.98. Found: C, 64.51; H, 3.77; N, 14.84.

### Characterisation of (E)-2-((4-((1–(4-nitrophenyl)-1H-1,2,3-triazol-4-yl)methoxy)benzylidene)amino)isoindoline-1,3-dione (8c)

This compound was obtained as pale yellow solid in 88% yield, mp: 270–271 °C. IR (υ, cm^−1^): 1550 (C = C), 1680 (C = N), 1715 (C = O), 2870 (CH-Al), 3030 (CH-Ar). ^1^H NMR (400 MHz, DMSO-d_6_) δ_H_ = 5.29 (*s*, 1.8H, OC**H_2_**), 5.31 (*s*, 0.2H, OC**H_2_**), 7.24 (dd, 2H, *J* = 4 Hz, *J* = 8 Hz, Ar-**H**), 7.40 (*t*, 2H, J = 4 Hz, Ar-**H**), 7.80–7.90 (*m*, 8H, Ar-**H**), 8.88 (*s*, 1H, C**H**-1,2,3-triazole), 9.04 (*s*, 0.7H, **H**C = N), 9.82 (*s*, 0.3H, **H**C = N). ^13^C NMR (100 MHz, DMSO-d_6_): δ_C_ = 61.71 (O**C**H_2_); 115.89, 122.45, 123.99, 124.16, 126.72, 130.57, 132.88, 134.80, 135.19, 135.73, 136.49, 144.26, 161.41, 165.00 (Ar-**C**, **C = N**, **C = O**). Calculated for: C_24_H_16_N_6_O_5_: C, 61.54; H, 3.44; N, 17.94. Found: C, 61.25; H, 3.54; N, 17.78.

### Characterisation of (E)-2-((4-((1–(3-fluoro-4-methylphenyl)-1H-1,2,3-triazol-4-yl)methoxy)benzylidene)amino) Isoindoline-1,3-dione (8d)

This compound was obtained as pale yellow solid in 87% yield, mp: 255–256 °C. IR (υ, cm^−1^): 1560 (C = C), 1660 (C = N), 1710 (C = O), 2850 (CH-Al), 3060 (CH-Ar). ^1^H NMR (400 MHz, DMSO-d_6_) δ_H_ = 2.28 (*s*, 3H, C**H_3_**), 5.32 (*s*, 2H, OC**H_2_**), 7.20 (d, 2H, *J* = 8 Hz, Ar-**H**), 7.49 (*t*, 2H, *J* = 4 Hz, Ar-**H**), 7.72–7.85 (*m*, 4H, Ar-**H**), 7.88–7.94 (*m*, 3H, Ar-**H**), 8.97 (*s*, 1H, C**H**-1,2,3-triazole), 9.10 (*s*, 1H, **H**C = N). ^13^C NMR (100 MHz, DMSO-d_6_): δ_C_ = 28.56 (**C**H_3_); 61.34 (O**C**H_2_); 115.77, 121.31, 123.57, 125.09, 126.82, 128.46, 130.74, 133.15, 134.64, 135.82, 136.82, 143.61, 160.62, 161.89, 165.37 (Ar-**C**, **C = N**, **C = O**). Calculated for: C_25_H_18_FN_5_O_3_: C, 65.93; H, 3.98; N, 15.38. Found: C, 65.79; H, 3.91; N, 15.46.

### Characterisation of (E)-2-((4-((1–(2-fluorophenyl)-1H-1,2,3-triazol-4-yl)methoxy)benzylidene)amino)isoindoline-1,3-dione (8e)

This compound was obtained as pale yellow solid in 86% yield, mp: 248–249 °C. IR (υ, cm^−1^): 1565 (C = C), 1690 (C = N), 1725 (C = O), 2915 (CH-Al), 3045 (CH-Ar). ^1^H NMR (400 MHz, DMSO-d_6_) δ_H_ = 5.37 (*s*, 2H, OC**H_2_**), 7.25 (d, 2H, *J* = 8 Hz, Ar-**H**), 7.87–8.00 (*m*, 9H, Ar-**H**), 8.30 (*s*, 1H, Ar-**H**), 9.10 (*s*, 1H, C**H**-1,2,3-triazole), 9.12 (*s*, 1H, **H**C = N). ^13^C NMR (100 MHz, DMSO-d_6_): δ_C_ = 61.63 (O**C**H_2_); 115.83, 120.78, 122.46, 123.91, 130.54, 130.58, 132.37, 135.40, 136.54, 160.82, 161.35, 165.07 (Ar-**C**, **C = N**, **C = O**). Calculated for: C_24_H_16_FN_5_O_3_: C, 65.30; H, 3.65; N, 15.87. Found: C, 65.53; H, 3.53; N, 15.72.

### Characterisation of ethyl (E)-4–(4-((4-(((1,3-dioxoisoindolin-2-yl)imino) methyl)phenoxy)methyl)-1H-1,2,3-triazol-1-yl)benzoate (8f)

This compound was obtained as pale yellow solid in 88% yield, mp: 224–225 °C. IR (υ, cm^−1^): 1540 (C = C), 1690 (C = N), 1725 (C = O), 2940 (CH-Al), 3050 (CH-Ar). ^1^H NMR (400 MHz, DMSO-d_6_) δ_H_ = 1.17 (*t*, 3H, *J* = 4 Hz, J = 8 Hz, C**H_3_**), 4.37–4.52 (*q*, 2H, C**H_2_**CH_3_), 5.34 (*s*, 2H, OC**H_2_**), 7.28 (d, 2H, *J* = 8 Hz, Ar-**H**), 7.43 (*t*, 2H, *J* = 4 Hz, Ar-**H**), 7.72–7.79 (*m*, 4H, Ar-**H**), 7.88–7.95 (*m*, 4H, Ar-**H**), 9.03 (*s*, 1H, C**H**-1,2,3-triazole), 9.15 (*s*, 1H, **H**C = N). ^13^C NMR (100 MHz, DMSO-d_6_): δ_C_ = 15.31 (**C**H_3_); 56.35 (**C**H_2_CH_3_); 61.56 (O**C**H_2_); 115.75, 117.81, 121.36, 123.70, 124.43, 125.06, 129.45, 130.42, 131.71, 133.69, 135.35, 144.65, 160.56, 161.39, 165.77 (Ar-**C**, **C = N**, **C = O**). Calculated for: C_27_H_21_N_5_O_5_: C, 65.45; H, 4.27; N, 14.13. Found: C, 65.29; H, 4.35; N, 14.27.

### Characterisation of (E)-2-((4-((1–(3,4-dichlorophenyl)-1H-1,2,3-triazol-4-yl)methoxy)benzylidene)amino)isoindoline-1,3-dione (8 g)

This compound was obtained as pale yellow solid in 87% yield, mp: 266–267 °C. IR (υ, cm^−1^): 1555 (C = C), 1680 (C = N), 1720 (C = O), 2935 (CH-Al), 3070 (CH-Ar). ^1^H NMR (400 MHz, DMSO-d_6_) δ_H_ = 5.31 (*s*, 1.4H, OC**H_2_**), 5.34 (*s*, 0.6H, OC**H_2_**), 7.24 (dd, 2H, *J* = 4 Hz, *J* = 8 Hz, Ar-**H**), 7.42 (dd, 1H, *J* = 4 Hz, *J* = 8 Hz, Ar-**H**), 7.51–7.59 (*m*, 2H, Ar-**H**), 7.79–7.88 (*m*, 6H, Ar-**H**), 8.74 (*s*, 1H, C**H**-1,2,3-triazole), 9.07 (*s*, 0.7H, **H**C = N), 9.83 (*s*, 0.3H, **H**C = N). ^13^C NMR (100 MHz, DMSO-d_6_): δ_C_ = 61.51, 61.66 (O**C**H_2_); 115.74, 115.80, 117.59, 117.75, 123.89, 126.14, 126.56, 130.50, 130.60, 135.36, 135.73, 143.44, 153.38, 155.37, 160.70, 161.43, 163.38, 165.03, 165.04, 165.06 (Ar-**C**, **C = N**, **C = O**). Calculated for: C_24_H_15_Cl_2_N_5_O_3_: C, 58.55; H, 3.07; N, 14.23. Found: C, 58.74; H, 3.15; N, 14.12.

### Characterisation of (E)-2-((4-((1–(4-acetylphenyl)-1H-1,2,3-triazol-4-yl)methoxy)benzylidene)amino)isoindoline-1,3-dione (8h)

This compound was obtained as pale yellow solid in 88% yield, mp: 275–276 °C. IR (υ, cm^−1^): 1540 (C = C), 1690 (C = N), 1730 (C = O), 2930 (CH-Al), 3080 (CH-Ar). ^1^H NMR (400 MHz, DMSO-d_6_) δ_H_ = 2.24 (*s*, 3H, C**H_3_**), 5.32 (*s*, 2H, OC**H_2_**), 7.24 (d, 2H, *J* = 8 Hz, Ar-**H**), 7.40 (*t*, 2H, *J* = 4 Hz, Ar-**H**), 7.70–7.81 (*m*, 4H, Ar-**H**), 7.89–7.98 (*m*, 4H, Ar-**H**), 9.06 (*s*, 1H, C**H**-1,2,3-triazole), 9.17 (*s*, 1H, **H**C = N). ^13^C NMR (100 MHz, DMSO-d_6_): δ_C_ = 24.56 (**C**H_3_); 61.73 (O**C**H_2_); 115.63, 116.96, 119.13, 121.65, 124.65, 125.34, 128.69, 130.25, 131.45, 134.26, 136.09, 143.36, 160.72, 161.75, 165.23 (Ar-**C**, **C = N**, **C = O**). Calculated for: C_26_H_19_N_5_O_4_: C, 67.09; H, 4.11; N, 15.05. Found: C, 67.27; H, 4.17; N, 15.14.

### Characterisation of (2S,3S,4R,5S,6S)-2-(acetoxymethyl)-6–(4-((4-((E)-((1,3-dioxoisoindolin-2-yl)imino)methyl)phenoxy)methyl)-1H-1,2,3-triazol-1-yl)tetrahydro-2H-pyran-3,4,5-triyl triacetate (10a)

This compound was obtained as white pellets in 91% yield, mp: 166–167 °C. IR (υ, cm^−1^): 1580 (C = C), 1660 (C = N), 1735 (C = O), 2830 (CH-Al), 3070 (CH-Ar). ^1^H NMR (400 MHz, DMSO-d_6_) δ_H_ = 1.70, 1.90, 1.94, 1.96 (4s, 12H, 4 x C**H_3_**CO), 4.01–4.11 (*m*, 2H, **H-6**, **H-6′)**, 4.32 (ddd, 1H, *J* = 4 Hz, *J* = 8 Hz, *J* = 12 Hz, **H-5**), 5.14 (dd, 1H, *J* = 4 Hz, *J* = 8 Hz, **H-3**), 5.21 (*s*, 1.30H, OC**H_2_**), 5.24 (*s*, 0.70H, OC**H_2_**), 5.49 (*t*, 1H, *J* = 8 Hz, **H-4**), 5.62 (dd, 1H, *J* = 4 Hz, *J* = 8 Hz, **H-2**), 6.32 (d, 1H, *J* = 8 Hz, **H-1**), 7.16 (dd, 2H, *J* = 4 Hz, *J* = 8 Hz, Ar-**H**), 7.76–7.86 (*m*, 6H, *J* = 8 Hz, Ar-**H**), 8.53 (*s*, 1H, C**H**-1,2,3-triazole), 9.03 (*s*, 0.65H, **H**C**=**N), 9.80 (*s*, 0.35H, **H**C**=**N). ^13^C NMR (100 MHz, DMSO-d_6_): δ_C_ = 20.34, 20.71, 20.85, 20.99 (4 x **C**H_3_CO); 61.55 (O**C**H_2_); 62.19 (**C**-5); 67.95 (**C**-6); 70.57 (**C**-4); 72.64 (**C**-3); 73.81 (**C**-2); 84.36 (**C**-1); 115.74, 115.82, 123.90, 124.44, 124.50, 126.62, 130.41, 130.48, 130.52, 132.32, 135.39, 143.35, 143.56, 160.90, 161.29, 163.26, 165.08 (Ar-**C**, **C = N**, **C = O**), 169.06, 169.96, 170.17, 170.66 (CH_3_**C**=O). Calculated for: C_32_H_31_N_5_O_12_: C, 56.72; H, 4.61; N, 10.34. Found: C, 56.53; H, 4.69; N, 10.46.

### Characterisation of (2S,3R,4R,5S,6S)-2-(acetoxymethyl)-6–(4-((4-((E)-((1,3-dioxoisoindolin-2-yl)imino)methyl)phenoxy)methyl)-1H-1,2,3-triazol-1-yl)tetrahydro-2H-pyran-3,4,5-triyl triacetate (10b)

This compound was obtained as white pellets in 90% yield, mp: 175–176 °C. IR (υ, cm^−1^): 1555 (C = C), 1670 (C = N), 1730 (C = O), 2890 (CH-Al), 3050 (CH-Ar). ^1^H NMR (400 MHz, DMSO-d_6_) δ_H_ = 1.71, 1.88, 1.92, 2.12 (4s, 12H, 4 x C**H_3_**CO), 3.98 (dd, 1H, *J* = 4 Hz, *J* = 12 Hz, **H-6)**, 4.09 (dd, 1H, *J* = 4 Hz, *J* = 12 Hz, **H-6′**), 4.51 (*t*, 1H, *J* = 4 Hz, **H-5**), 5.24 (*s*, 2H, OC**H_2_**), 5.40 (dd, 2H, *J* = 4 Hz, *J* = 8 Hz, **H-3**, **H-4**), 5.57 (dd, 1H, *J* = 4 Hz, *J* = 8 Hz, **H-2**), 6.34 (d, 1H, *J* = 8 Hz, **H-1**), 7.18 (d, 2H, *J* = 8 Hz, Ar-**H**), 7.79–7.85 (*m*, 6H, *J* = 8 Hz, Ar-**H**), 8.48 (*s*, 1H, C**H**-1,2,3-triazole), 9.81 (*s*, 1H, **H**C**=**N). ^13^C NMR (100 MHz, DMSO-d_6_): δ_C_ = 20.40, 20.79, 20.88, 20.97 (4 x **C**H_3_CO); 61.55 (O**C**H_2_); 62.04 (**C**-5); 67.77 (**C**-6); 68.18 (**C**-4); 70.89 (**C**-3); 73.48 (**C**-2); 84.77 (**C**-4); 115.75, 115.81, 116.37, 124.72, 124.98, 126.78, 130.40, 130.63, 132.33, 134.72, 143.19, 143.62, 160.82, 163.28, 164.92 (Ar-**C**, **C = N**, **C = O**), 169.10, 170.07, 170.56, 170.59 (CH_3_**C**=O). Calculated for: C_32_H_31_N_5_O_12_: C, 56.72; H, 4.61; N, 10.34. Found: C, 56.56; H, 4.55; N, 10.45.

## Biological evaluation studies

### Antiviral assay

The effect of target chemical treatments on SARS-CoV-2 viral load (SARS-CoV-2 isolate EGY/WAT-2 VACCERA) was assessed by the Real-Time PCR test to detect SARS-CoV-2 viral RNA^52^^,^^53^. Total RNA was extracted according to the instructions using the genesig® Coronavirus SARS-CoV-2 Real-Time PCR Assay kit (Primer design TM Ltd, Southampton, United Kingdom). All procedures are depicted in the supplementary section.

### Cytotoxicity evaluation using a viability assay

The cytotoxic activity was assessed using the 3–(4,5-dimethylthiazol-2-yl)-2,5-diphenyl tetrazolium bromide (MTT) colorimetric assay as reported previously. In brief, the tumour cell lines were suspended in medium at concentration 5 × 104 cell/well in Corning® 96-well tissue culture plates and then incubated for 24 h. The tested compounds with concentrations ranging from 0 to 50 μg/ml were then added into 96-well plates (six replicates) to achieve different conc. for each compound. Six vehicle controls with media or 0.5% DMSO were run for each 96 well plate as a control. After incubating for 24 h, the numbers of viable cells were determined by the MTT test^54–56^.

### SARS-CoV-2-M^Pro^ inhibition assay

The COV2-SARS-CoV-2 protease enzyme assay^46^^,^^57–59^ was described in the manufacturing protocol (BPS Bioscience)^60^. The methodology is described in the supplementary section.

### Molecular docking

The interaction of the designated compounds with SARS-CoV‑2 M^pro^ Enzyme] were performed with the aid of MOE software^61^. Also, we used the graphical user interface program autodock software as a complementary tool^62^. The protein crystal structure in combination with Bipyridine benzonitrile was extracted from the RCSB PDB^63^. Preparation of the protein was performed over two steps using Maestro 8.0’s ‘protein preparation wizard. To vert any electronic clash in the protein, the energy of the crystal structure was minimised using OPLS 2005 force field. The binding site was assigned as indicated by the native ligand, and the receptor grid was created using “Grid Generation tool”. Finally, a single best pose was generated as the output for the tested ligands.

## Results and discussions

### Chemistry

**Scheme 1.**
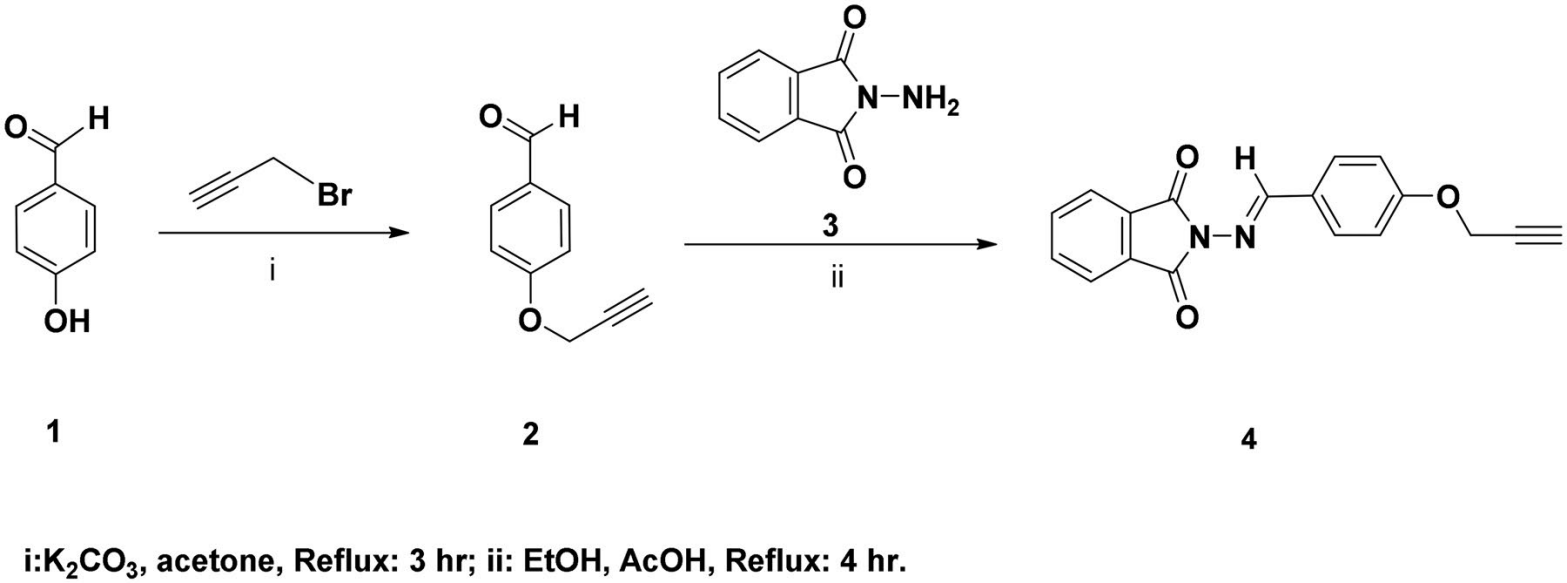
Synthesis of phthalimide Schiff base with terminal alkyne tether **4**.

**Scheme 2.**
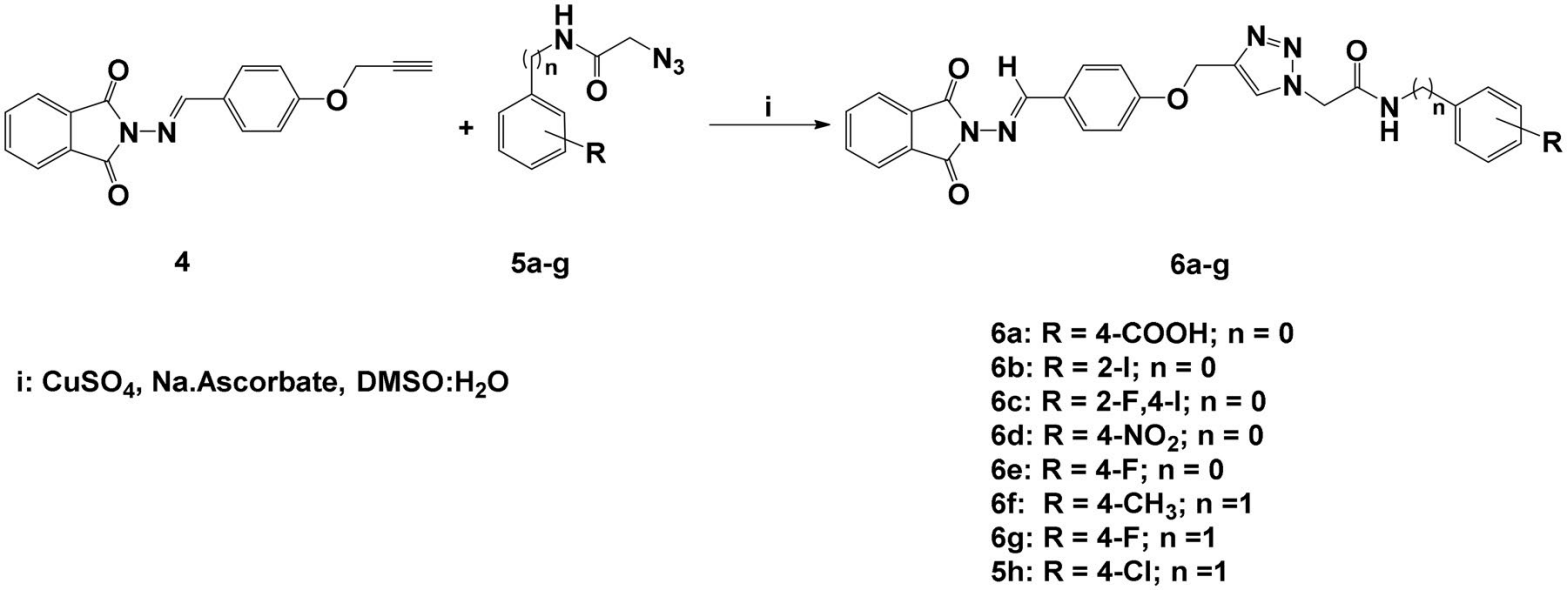
Synthesis of 1,2,3-triazole-phthalimide hybrids with phenylacetamide tethers **6a-g**.

**Scheme 3.**
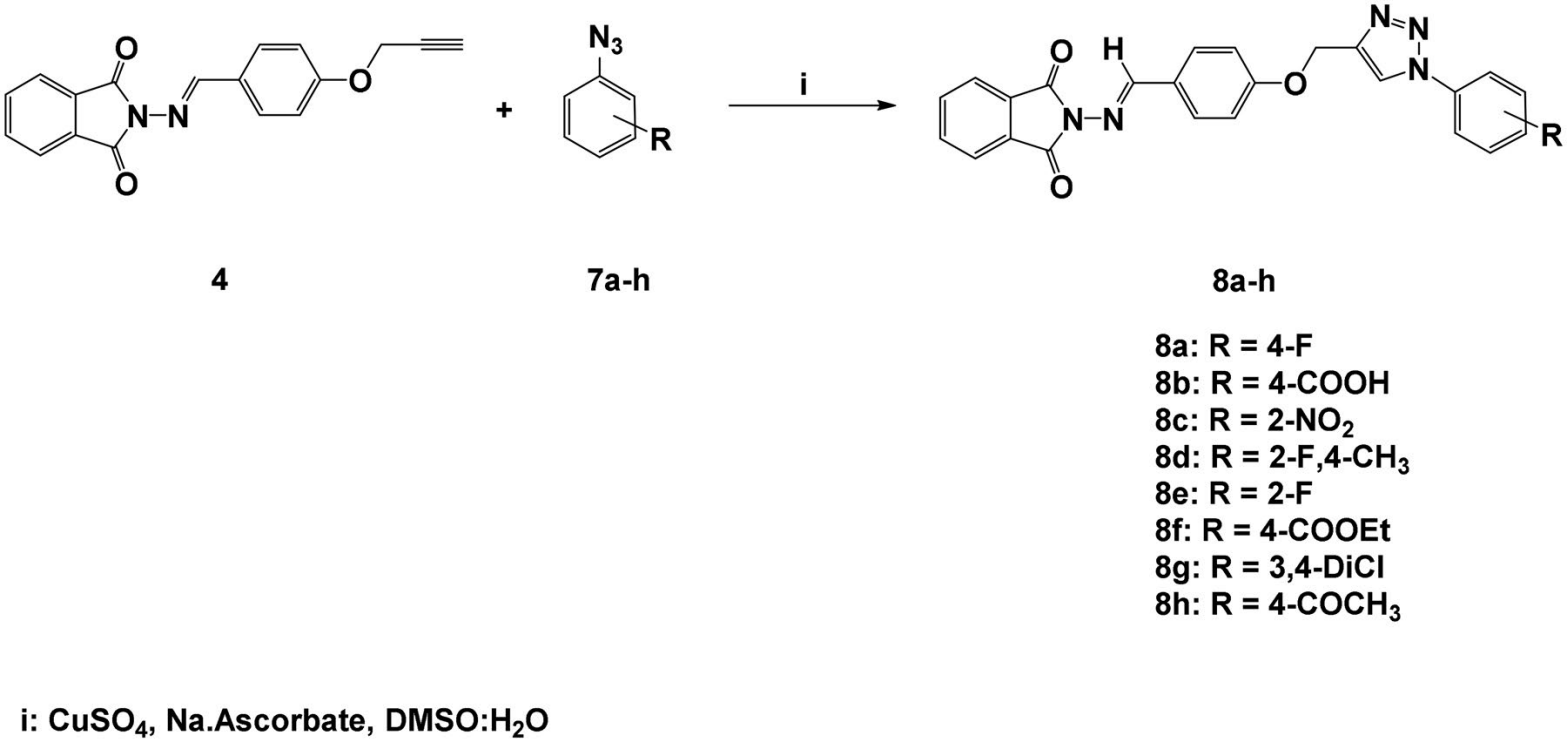
Synthesis of 1,2,3-triazole-phthalimide hybrids bearing aromatic ring **8a-h**.

**Scheme 4.**
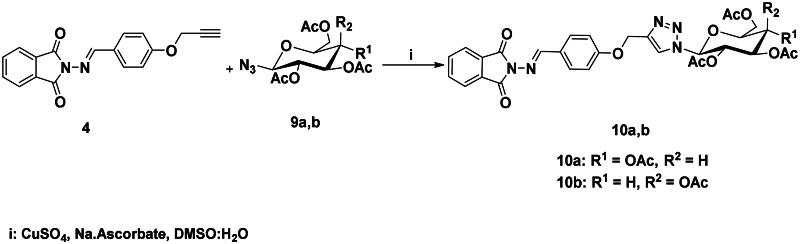
Synthesis of 1,2,3-triazole-phthalimide hybrids bearing glycosyl moiety **10a-b**.

The synthetic route adopted for the synthesis of the targeted phatamide-1,2,3-triazole molecular conjugates was outlined in Schemes 1–4. The precursor phthalimide-based azomethine linkage and alkyne side chain **3** was successfully synthesised in 92% yield through the thermal condensation of 2-amino­isoindoline-1,3-dione (**3**) and 4-(prop-2-yn-1-yloxy)benzaldehyde (**2**) in the presence of catalytic amount of acetic acid for 4 h. According to our previously published work^46^, the alkyne 2 was synthesised by propargylation of 4-hydroxybezaldehyde 1 in refluxing acetone with potassium carbonate as a basic catalyst (Scheme 1).

The chemical structure of the synthesised Schiff base **4** was elucidated by spectroscopic methods. Thus, its IR spectrum was in obvious with the structure. It showed the disappearance of the carbonyl aldehyde and amino groups of the starting materials **2** and **3**, respectively, which confirmed their condensation in the formation of the resulted azomethine group (C = N). The ^1^H NMR spectrum also supported the condensation reaction by the absence of amino (N**H_2_**) and aldehyde (C**H**O) protons, and presence of the distinct singlet at δ_H_ 9.11 ppm attributed to the imine proton (**H**C = N). The spectrum also revealed two characteristic singlets at δ_H_ 3.65 and δ_H_ 4.91 ppm assigned to the ≡C**H** and OC**H_2_** protons, respectively. Extra aromatic protons were observed in the aromatic area. Moreover, its ^13^C NMR spectrum showed the appearance of signals at δ_C_ 160.52–165.02 ppm assigned to the imine (**C = N**) and phthalimide carbonyl amide (**C = O**) carbons, respectively. The diagnostic acetylenic carbons (-**C**≡**C**-) were observed at δ_C_ 79.15 and 79.27 ppm. All remaining carbons were recorded at their expected chemical shift (See experimental section).

Using the previously reported approach^34^, focused substituted anilines were effectively converted to the desired phenylacetamide azides **5a-h** through their alkylation with chloroacetyl chloride followed by azidolysis reaction. Within the scope on the molecular hybridisation, the desired molecular hybrids **6a-h** were obtained with good to excellent yields (87–90%); by the construction of the 1,2,3-triazole via the copper-catalyzed azide-alkyne cycloaddition (CuAAC) between the phthalimide Schiff base bearing alkyne side chain **4** and the synthesised azides **5a-h** in the presence of copper sulphate and sodium ascorbate as catalysts in a mixture of DMSO:H_2_O as solvent at 80 °C for 6–8 h (Schemes 2 and 3).

The success of the cycloaddition reaction was confirmed by the spectral data of the resulting click adducts **6a-g**. Their IR spectra showed clearly the disappearance of the acetylenic groups (C≡C and ≡C-H) of their precursor **4**, which confirmed its involvement on the formation of 1,2,3-triazole ring. The investigation of their ^1^H NMR spectra revealed the presence of a diagnostic singlet at around δ_H_ 8.21–8.33 ppm attributed to the characteristic H-5-triazolyl ring. The amidic protons (CON**H**) were recorded as singlets at δ_H_ 9.03–11.12 ppm, where the methylene protons (OC**H_2_**, NC**H_2_** and NHC**H_2_** were observed at δ_H_ 4.30–5.47 ppm. The remaining protons were resonated at their respected area and listed in the experimental section. The analysis of the ^13^C NMR data showed clearly disappearance Sp-carbons and the appearance of the new aliphatic carbon signals (O**C**H_2_, N**C**H_2_ and NH**C**H_2_) between δ_C_ 42.12–61.88 ppm. Additional aromatic carbons resonating in their respected chemical shifts (See experimental section).

Furthermore, we have expanded our efforts to synthesise several new phthalimide-1,2,3-triazole hybrids encapsulating lipophilic aromatic rings **8a-h** (87–90% yield) utilising the optimised click synthesis outlined above (CuSO_4_, Na-ascorbate; H_2_O:DMSO), as shown in Scheme 3. The synthetic strategy started with the synthesis of aromatic azide building blocks **7a-h** by diazotisation reactions of their corresponding aniline derivatives as previously reported in our work^37^.

The structures of compounds **8a-h** were in obvious of their IR spectra, which confirmed the participation of the acetylenic groups of the alkyne **4** on the formation of the triazole core. The formation of such ring in compounds **8a-h** was also supported by their ^1^H NMR spectra which displayed the resonance of the distinct H-5-triazolyl proton as a singlet around δ_H_ 8.74–9.10 ppm. The spectra also recorded signals attributed to the methylene protons (OC**H_2_**) at δ_H_ 5.29–5.37 ppm. Additional aromatic protons of the phenyl rings were observed in the aromatic area and are detailed in the experimental section. No Sp-carbons were recorded in their ^13^C NMR data which evidenced the success of the cycloaddition reaction. All remaining carbons were listed in the experimental section. Owing to the fact that the molecular hybridisation strategy has attracted continuing interest for drug developments, it was interested to vary the azide structure to increase the synergetic effect of the resulted click candidates. Thus, click synthesis of a focused 1,2,3-triazole-phthalimide harbouring glycosyl moieties **10a-b** was also carried out in this study. The click ligation of the same precursor alkyne **4** with glycosyl azides **9a-b** under the previously adopted copper 1,3-dipolar cycloaddition reaction conditions gave selectively the targeted 1,4-disubstituted-1,2,3-triazoles based phthalimide-N-glycoside molecular hybrids in 90–91% yield (Scheme 4).

The structure elucidation of the resulted click adducts **10a-b** were established FT-IR, ^1^H NMR, ^13^C NMR spectroscopic methods, which evidenced the success of the click synthesis by the appearance of a distinct doublet at δ_H_ 6.32 and 6.34 ppm with J_1′,2′_ value equal to 8 Hz attributed to the glycosyl anomeric proton (**H-1**) and confirming the β-configuration. The recorded anomeric protons were correlated with their respected carbons **C-1** resonating at δ_C_ 84.36 and 84.77 ppm supporting the β-anomers. New singlets were observed in the aliphatic regions in their ^1^H NMR spectra (δ_H_ 1.70–2.12 ppm) and correlated with their carbons (δ_C_ 20.34–20.99 ppm) in the^13^C NMR spectra, these signals were assigned to the diagnostic acetoxy groups (OAc). Furthermore, the triazolyl H-5 were observed at δ_H_ 8.48–8.53 ppm confirming the ring closure and cycloaddition reaction. All remaining protons and carbons were recorded at their respect chemical shifts (See experimental section).

### Evaluation of antiviral activity against SARS-CoV-2

At the outset of our antiviral investigations, we tested the ability of the synthesised molecules to inhibit SARS CoV-2 in infected Vero E6 cells in terms of (% of inhibition) compared to reference drug. We utilised two concentrations to perform our assay, 1 and 10 uM. In addition, we tested the cytotoxic effect of our molecules in Vero E6 cells to correlate antiviral activity and cytotoxicity and ensure their low or no cytotoxic effects on the cells. The data from these assays are summarised in Table 1. At 1 uM concentration, molecules **6a** and **8e** with 4-carboxyphenyl and 2-fluoroophenyl side chain, respectively, showed comparable activity to Remdesivir (87.82 and 83.58 vs. 92.72%). On the other hand, the other derivatives showed moderate to weak % of inhibition as indicated by their API values.

**Table 1.** Antiviral activity of novel phthalimide-based triazole inhibitors by propagation in Vero E6 cells.

Comp.ID	Scaffold	SARS-CoV-2 antiviral activity (%)		**(API)** ^a^	**CT/A** ^b^	Vero E6 cytotoxicity (mM)
	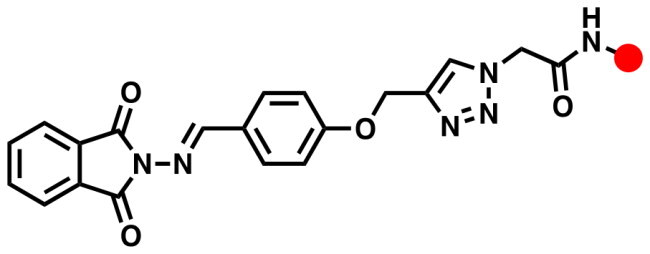	**1 mM**	**10 mM**			
**6a**	4-carboxyphenyl	87.82	94.96	**0.98**	**9.95**	7.6 *±* 99.5
**6b**	2-iodophenyl	68.45	94.37	0.97	6.50	4.3 *±* 65.0
**6c**	2-fluoro-4-iodophenyl	67.11	93.01	0.96	2.99	2.3 *±* 29.9
**6d**	4-nitrophenyl	69.74	94.82	0.98	7.05	5.4 *±* 70.5
**6e**	4-fluorophenyl	74.74	92.87	**0.96**	**10.80**	8.3 *±* 108
**6f**	4-methylbenzyl	72.16	89.33	0.92	3.17	2.4 *±* 31.7
**6g**	4-fluorobenzyl	82.15	94.29	**0.97**	**9.75**	7.4 *±* 97.5
**6h**	4-chlorobenzyl	80.19	92.29	0.95	6.02	4.6 *±* 60.2
	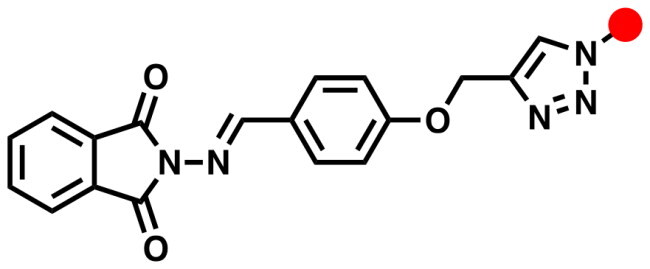	**1 mM**	**10 mM**			
**8a**	4-fluorophenyl	79.35	93.70	**0.96**	**12.7**	9.7 *±* 127.0
**8b**	4-carboxyphenyl	63.74	88.00	0.91	4.57	3.5 *±* 45.7
**8c**	p-nitrophenyl	81.13	93.34	0.96	8.62	6.6 *±* 86.2
**8d**	3-fluoro-4-methylphenyl	54.40	89.40	0.92	4.06	3.1 *±* 40.5
**8e**	2-fluorophenyl	83.58	95.16	0.98	6.59	5.0 *±* 65.9
**8f**	Ethyl benzoate	69.13	88.07	0.91	6.99	5.3 *±* 69.9
**8g**	3,4-dichlorophenyl	75.77	93.44	0.96	2.84	2.2 *±* 28.4
**8h**	4-acetylphenyl	77.71	93.79	0.96	4.28	3.3 *±* 42.8
**10a**	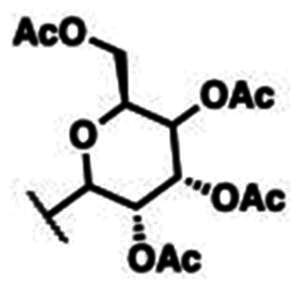	80.74	89.10	0.92	6.46	4.9 *±* 64.6
**10b**	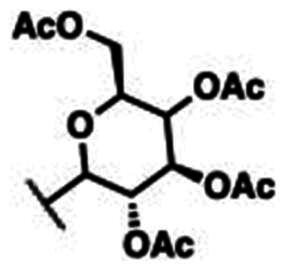	67.78	87.82	0.90	10.80	8.2 *±* 108.0
**Remdesivir**	NA	92.72	96.72	NA	4.31	3.3 *±* 43.1

^a^Antiviral potency index: It is the ratio of the inhibition % of the tested compounds to that of Remdesivir at 10 μM; 1 = Identical effect.

^b^Cytotoxicity/activity (10 mM) parameter.

At 10 uM, the majority of our derivatives started to show exceptional inhibition activities in comparison with Remdesivir. API parameter showed values ranging from 0.9 to 0.98 (Table 1). Compound **6a**, with 4-propionamidobenzoic acid side chain, showed the highest activity among the tested compounds and the better API value in comparison with Remdesivir (94.96% and 0.98). In order to ensure that any given activity of our compounds is not due to a potential toxicity to the tested cells, we introduced Vero E6 cells cytotoxicity as an additional parameter in our evaluation. To distinguish the performance of our molecules in both sides (antiviral activity and toxicity), we generated a parameter called CT/A parameter which compare the IC_50_ (in uM) of the tested molecule (include Remdesivir) with the antiviral activity maximum concentration (10 uM). Higher CT/A value implies that the tested molecule was safe to the cells during the assay, and it is applicable to increase the drug concentration to achieve 100% eradication of the virus. Compound **8a** bearing 4-fluorophenyl achieved the best CT/A parameter which represents (12.7). This value means that the safety margin of this compound represents 12.7-fold of its antiviral activity. Additionally, Remdesivir came in the 5^th^ place among the molecules that showed the lowest CT/A parameter (4.31). Among the tested compounds, **6a**, **6e**, **6 g**, and **8a** displayed the best combination between the antiviral activity and tolerability at 10 uM. Since derivatives **6e** and **8a** share the same side chain (4-fluorophenyl), we concluded that fluorene atom at that position may be essential for the antiviral activity of this scaffold.

**Figure 3.**
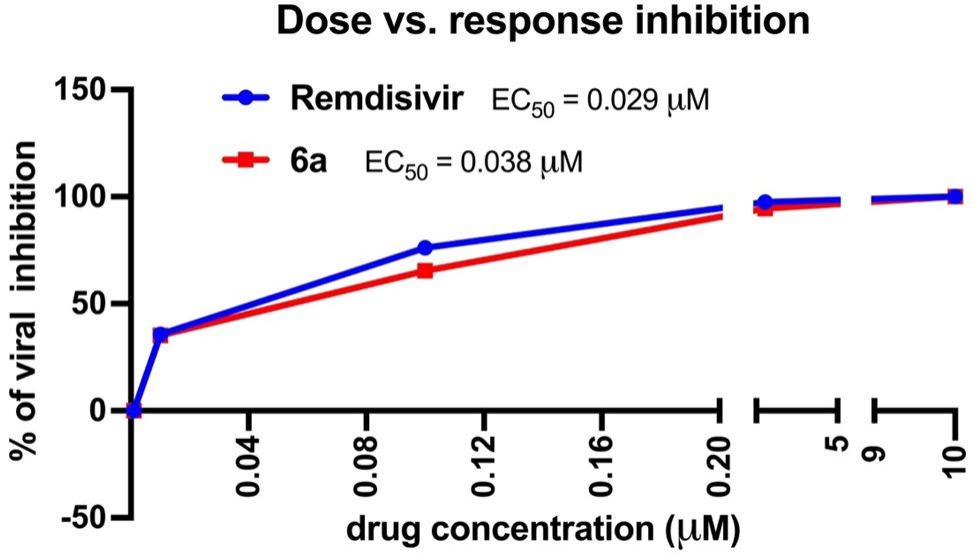
Dose starting from 10 uM of the tested compounds.

Then, we tested the effect of compound **6a** as a representative of the most active compounds in our series, at five different concentrations. We compared the effect of our compound with Remdesivir, the drug of choice for the treatment of COVID-19. The data are summarised in Figure 3.

The tested molecules (Remdesivir and **6a**) showed a dose–response inhibition of the virus with comparable EC_50_ values (0.029 and 0.039 uM). At a very low dose (0.01 uM) our compound showed ∼ 40% virus inhibition. This value represents 995 times the CT/A value of this compound. This data collectively indicated that this scaffold is a promising starting point to generate an effective treatment for SARS-CoV-2.

### SARS-CoV-2 M^pro^ inhibition assay

In our effort to detect the mechanism of action of our compounds, we tested the inhibition effect of SARS-CoV-2 M^pro^ by a designated derivative of our designed compounds. In several studies, the SARS-CoV-2 M^pro^ is the key protease of CoV-2 and was proved as one of the fundamental targets to impact this virus^64^^,^^65^. Many SARS-CoV-2 M^pro^ inhibitors have been reported that can covalently or non-covalently have been reported^66^. Some of the reported compounds carries the same triazol scaffold^67^. Such reports encouraged us to envision the impact of our molecule on SARS-CoV-2 M^pro^. We tested the effect of the *in vitro* M^pro^ inhibition effect of the selected compounds using MTT assay, and compared it with lopinavir, the non-specific M^pro^ inhibitor^68^. Results from this assay is summarised in Table 2.

**Table 2.** Inhibitory data of selected derivatives against SARS-CoV-2 M^pro^.

Comp. ID	Chemical structure	IC_50_ (mM)
**6a**	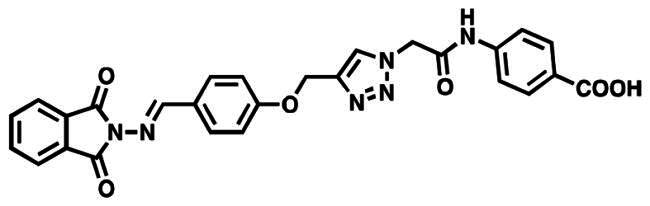	*112.8 ±* 5.75
**6 g**	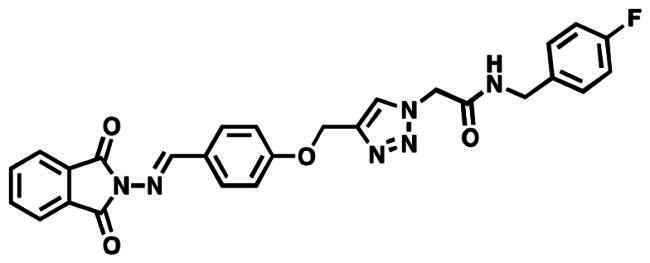	*90.09 ±* 4.59
**10a**	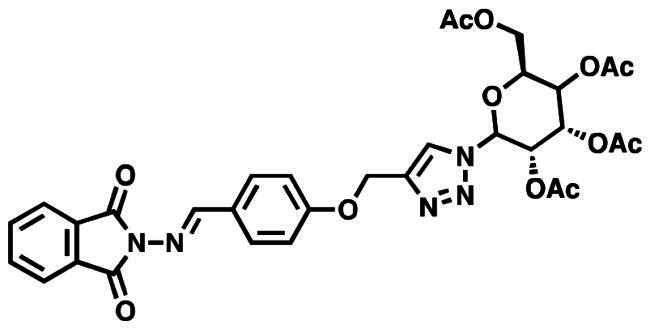	1088 ± 55.5
**Lopinavir**	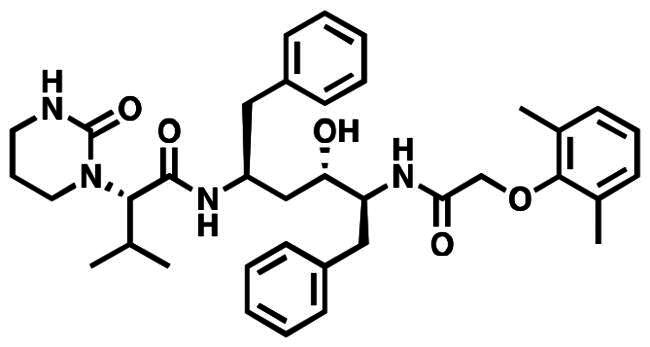	148.8 ± 7.48

Compounds **6a** and **6 g** showed IC_50_ values higher than Lopinavir, the non-specific M^pro^ inhibitor. Compound **6a** was significantly more active than the other tested molecules and of the reference molecule. However, none of the tested compounds showed an IC_50_ concentration comparable to its viral inhibitory effect (10 uM, Table 1). The results suggest that while the virus growth significantly inhibited by some of our compounds, the M^pro^ function did not highly altered. This observation suggesting that our molecules may have a mechanism of action independent of the M^pro^ target.

## Computational studies

### Molecular docking analysis

**Figure 4.**
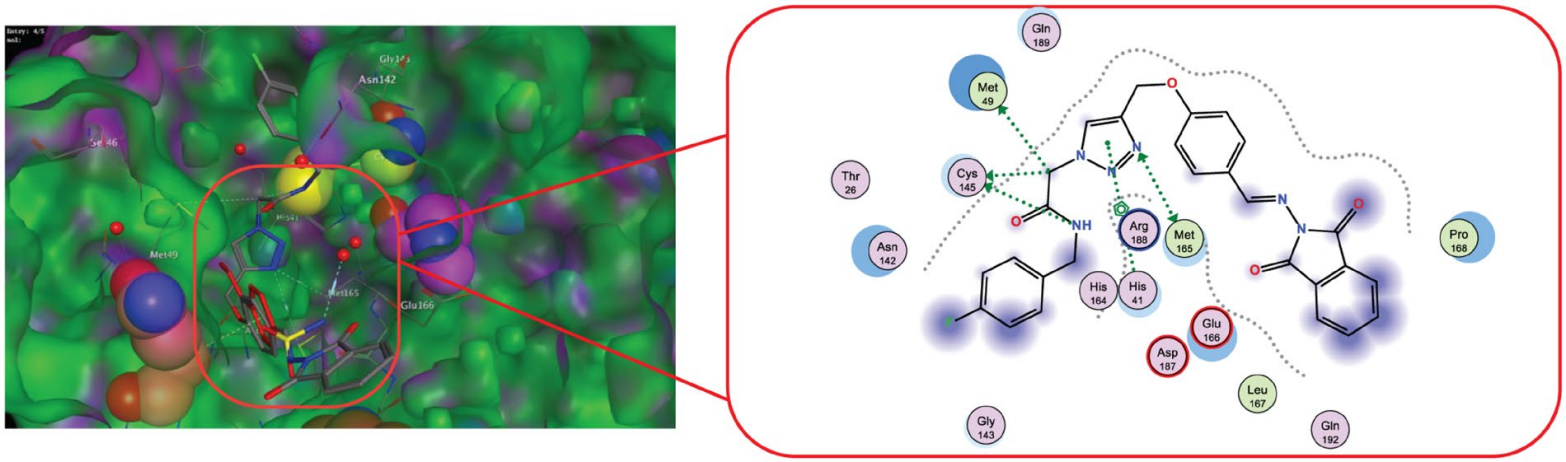
Docked poses of M^pro^ protease (PDB ID: 5R80) complexed with **Z18197050** and **6 g** compound with 2D interaction map for 6 g (right) and 3D interaction map of both aligned compounds (left).

The M^pro^ active site is located between two-barrel domains, (1^st^ amino acids 10–99, and 2^nd^ amino acids 100–182). The 3rd domain, which consists of residues 198 to 306, is responsible for protein dimerisation and the formation of an alpha helical bundle^50^. In addition, two important conserved residues, His41 and Cys145, create the catalytic dyad and dimerisation that complete the active site by bringing Ser161 of the second dimer protomer into proximity with Glu166 and promoting creation of the substrate specificity pocket and the oxyanion hole ^51^. In order to stabilise the molecule within the binding pocket, the phenoxy and triazole fragments interacted with the essential residues Gln189 and Met165, His41, and Arg188 via hydrogen bonds and the aromatic stacking effect. The catalytic Cys145 provided a stable hydrogen bonding with the middle amide moiety which might help in the stabilisation of these compounds within S1’ subsite. Reports state that the S2 subsite showed more flexibility than the others, preferring leucine or other hydrophobic residues while accepting smaller substituents in peptide-based inhibitors^57^. The majority of specific interaction of active analog occurs centrally through dual hydrogen bonding with Cys145-NH linker of amide, and Met165-n triazole, and a pattern of an aromatic ring formation - stacking interactions with Met165, which drives the terminal phthalimide component to near to S1’ site. Analysing the interaction of compound 6 g (the most prominent derivative in our series) showed no covalent interactions within the binding pocket. The majority of chemical moieties in this molecule contribute to the activity and tolerate the pocket with all catalytic sites, as shown in Figure 4.

## ADMET analysis

Then we used OSIRIS Property Explorer tool to estimate the adverse effects risk of designated analogues **6a**, **6 g,** and **10a**. These effects include tumorigenic effects and reproductive consequences. In addition, we investigated some drug-relevant characters, such as cLogP, LogS as an indication of the compounds solubility, drug-likeness, and overall drug-score^69^^,^^70^. We compared the results from our molecules with two drugs, GC-376 and Lopinavir. The results from these calculations are shown in Table 3. All the compounds showed an acceptable “predicted” solubility and cLogP values. On the other hand, none of our molecule showed any potential toxicity risks as indicated by the green colour code. In addition, the potential drug-likeness scores of the compound **6a** were superior to **6 g** and GC-376 and comparable to **lopinavir**. Chemically, our data showed that that tethering **COOH** or **F** as substituents to the peripheral phenyl group sustain a minimal danger of carcinogenic and mutagenic toxicity while appropriately boosting lipophilicity. Generally, the values of drug-score of the representative **6a** derivative (0.28) were less than compared to **GC-376** (0.37) and better than **Lopinavir** (0.17).

**Table 3.** Calculation of certain properties of compounds 6f, 6i, and 10a in comparison with reported drugs as predicted by OSIRIS Property Explorer and Molinspiration tools.

ID	Molecular properties				Druggability		Toxicity risks				%ABS
	TPSA	Mwt	Solubility	clogP	Druglikeness	Drug-score	M	T	I	R	
6a	156	524	−4.69	3.36	−6.57	0.28					55.18
6g	118	512	−4.81	3.74	−6.1	0.28					68.29
10a	204	677	−5.61	2.10	−6.32	0.18					38.62
GC-376	179	485	−2.75	−0.97	−28.75	0.37					47.24
Lopinavir	120	628	−6.13	4.85	7.64	0.17					67.60

https://www.organic-chemistry.org/prog/peo.

The calculation of %ABS was done using this equation ABS = 109−(0.345× TPSA). Green colour encodes no side effects will be reported.

Next, we performed a computational study to predict ADME properties of the synthesised molecules. We calculated several properties such as lipophilicity, absorption (% ABS), topological polar surface area (TPSA), and simple molecular descriptors represented by the Lipinski rule of five^55^^,^^71–73^. We used the Molinspiration online property calculation toolkit to perform our calculations^74^. Data from these calculations is summarised in Table 3. It is worth noting that the software used the Zhao equation (% ABS = 109−(0.345 × TPSA)) to calculate the percentage of absorption (% ABS)^75^. The predicted percentages of absorption for our compounds were ranging between 68 and 38%. In addition, the TPSA values located within the acceptable range as indicated in Table 3. Collectively, data from both calculations revealed that our molecules are most likely drug-like candidates^13^^,^^45^^,^^46^.

## Molecular descriptors–based SAR analysis

Next, we used the MOPAC quantum engine module implemented in the MOE software to perform quantum mechanical calculations for the SAR analysis of representative molecules from our series (**6a**; high activity, **6 g**; low activity, and **10a**; inactive). The energies of frontier orbitals typically have a significant impact on various chemical and pharmacological process properties. These characteristics provide details about the drugs’ acceptors and donators, and as a result, they provide details about how a charge transfer complex (CTC) forms ^21^^,^^72^. The Table 4 summarises our findings from these calculations. Electron cloud formed by the COOH group to compound **6a** is likely to contribute positively to the biological activity of this derivative. On the other hand, the analysis is different in the case of the other two molecules. The presence of another phenyl substitution (F) in case of **6 g** or polar nucleoside in the case of **10a** altered the calculations. Compounds **6 g** and **10a** showed slight or major difference in SCF energy and dipole effect, respectively. This data further confirms the impact of certain substitution on the activity of our compounds.

**Table 4.** Molecular mechanics parameters and molecular orbital spatial distribution and localisation for the HOMO-LUMO of representative compounds, **6a**, **6 g**, and **10a**.

Parameter	6a	6g	10a
**Scaffold**	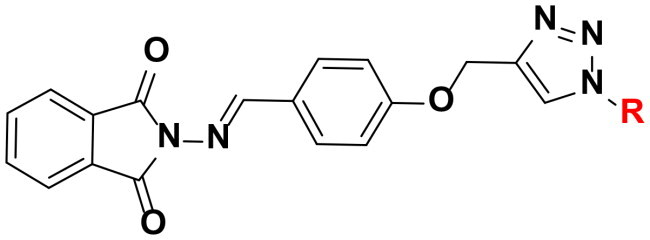		
**Active fragments**	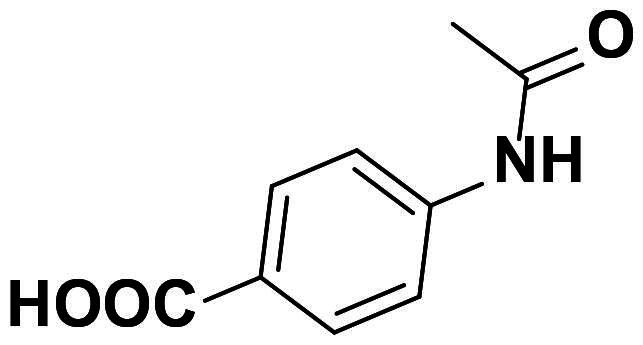	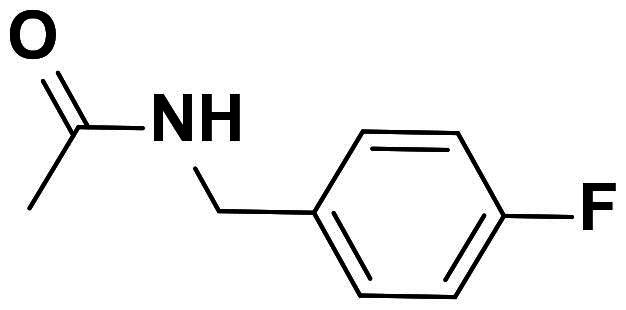	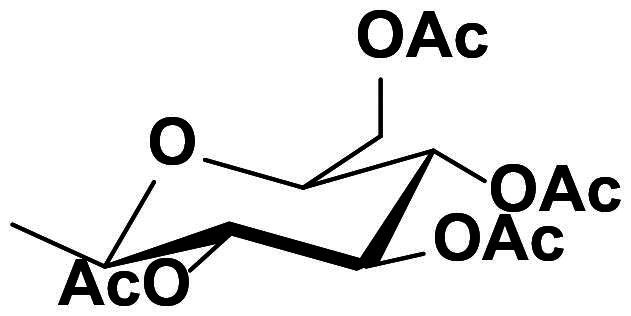
**Molecular orbitals**	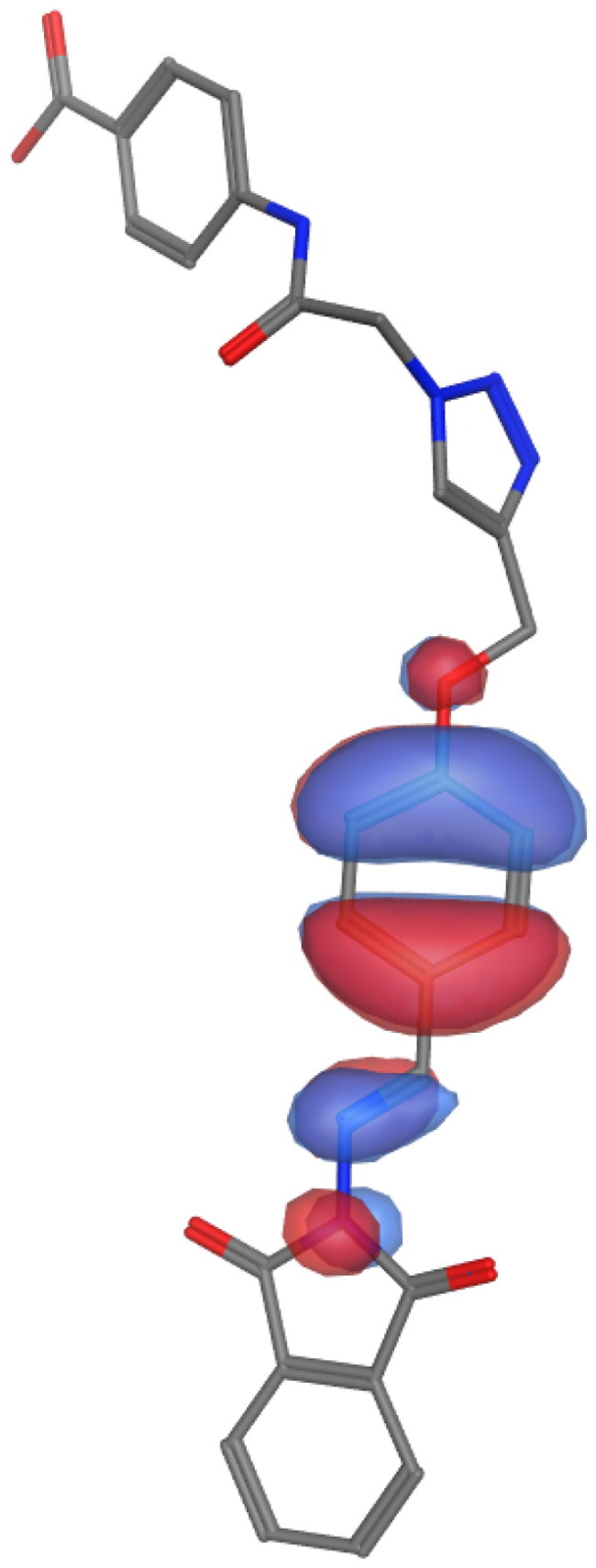	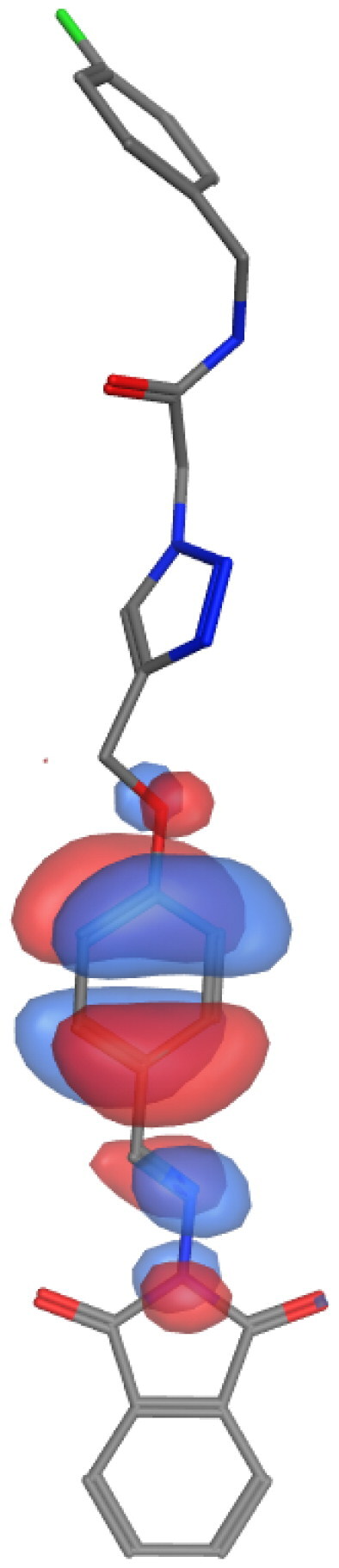	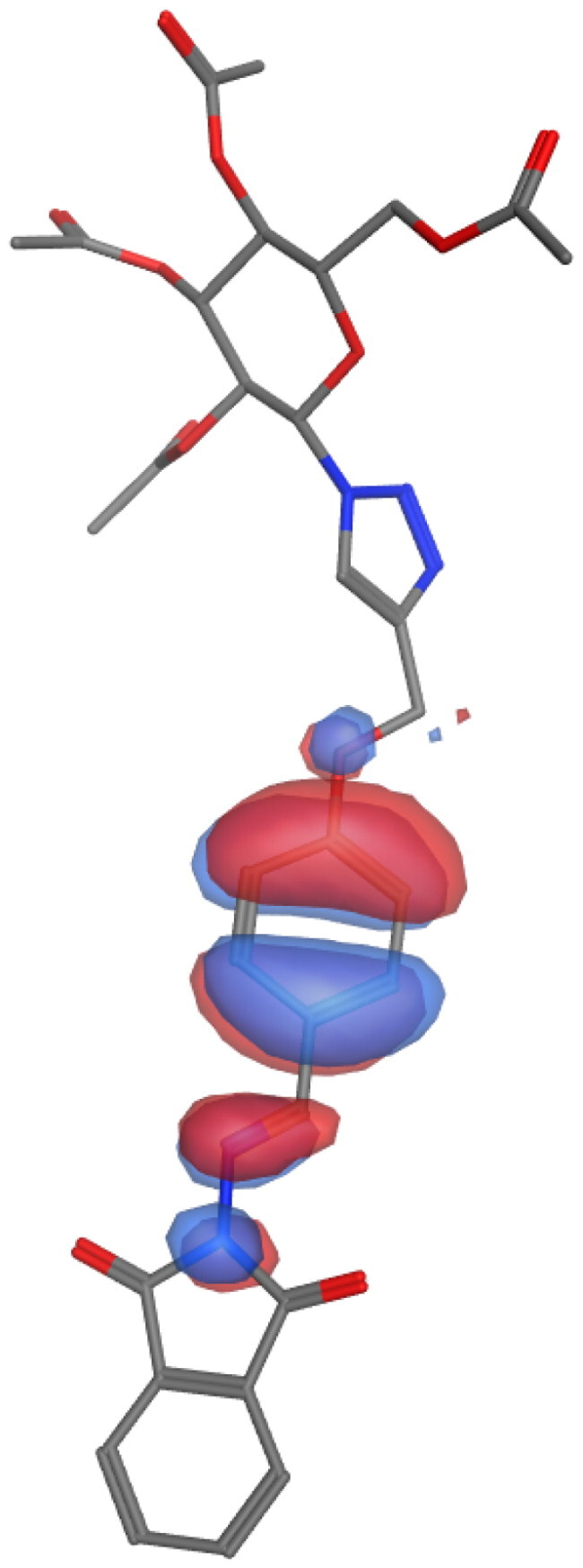
**SCF energy (kcal/mol)**	**−253**	**−247**	**−345**
**Dipole (D)**	**8.53**	**4.58**	**7.98**
**Electrons**	**194**	**190**	**256**
**EHOMO (eV)**	**−1.3**	**−1.2**	**−1.4**
**ELUMO (eV)**	**−8.91**	**−8.85**	**−9.04**
**Delta (eV)**	**7.63**	**7.60**	**7.70**

Molecular parameters are calculated by default from MOE program showing number of atoms, orbitals, electrons, SCF energy, dipole moment, and heat of formation. These values are reported for representative derivatives of high (**6a**), medium (**6 g**), and low (**10a**) activity.

## Conclusions

This current study details the synthesis of novel molecular conjugates incorporating phthalimide-1,2,3-triazole and assesses their effectiveness in combating SARS-CoV infection. We conducted initial investigations into the mechanism of action to elucidate the process of virus inhibition. The results revealed two highly potent analogues, **6a** and **6 g**, within the novel scaffold, demonstrating an impressive 94% growth inhibition against the SARS-CoV virus. *In vitro* inhibition data for M^pro^ suggested that our compounds might not exclusively target M^pro^, implying that the notable virus inhibition observed may be attributed to a different target. The successful development of a stable chemical scaffold with favourable pharmacokinetic properties holds significant promise for advancing antiviral drug discovery. Further studies are imperative to unravel the specific target of these molecules.

## Supplementary Material

Supplemental Material

## Data Availability

The authors confirm that the data supporting the findings of this study are available within the article and/or its supplementary materials.

## Sample availability

No Samples are available.
