# Supplementary material for: Exploring of N-phthalimide-linked 1,2,3-triazole analogues with promising ­anti-SARS-CoV-2 activity: synthesis, biological screening, and molecular modelling studies
Source: J Enzyme Inhib Med Chem. 2024 Jun 7;39(1):2351861. doi: 10.1080/14756366.2024.2351861 (PMC11164105; doi:10.1080/14756366.2024.2351861)
Supplement: Supplemental Material [file IENZ_A_2351861_SM6460.pdf]

Exploring of *N*-phthalimide-linked 1,2,3-triazole analogs with promising anti-SARS-CoV-2 activity: synthesis, biological screening, and molecular modeling studies

Ateyatallah Aljuhani,[1] Mosa Alsehli,[1] Mohamed A. Seleem,[2] Shaya Y. Alraqa,[1] Hany E. A. Ahmed,[2,*] Nadjet Rezki,[1,*] Mohamed R Aouad[1]

Supplementary Materials

1. Spectroscopic data of target compounds
2. Experimental detailed procedures

1. Chemistry

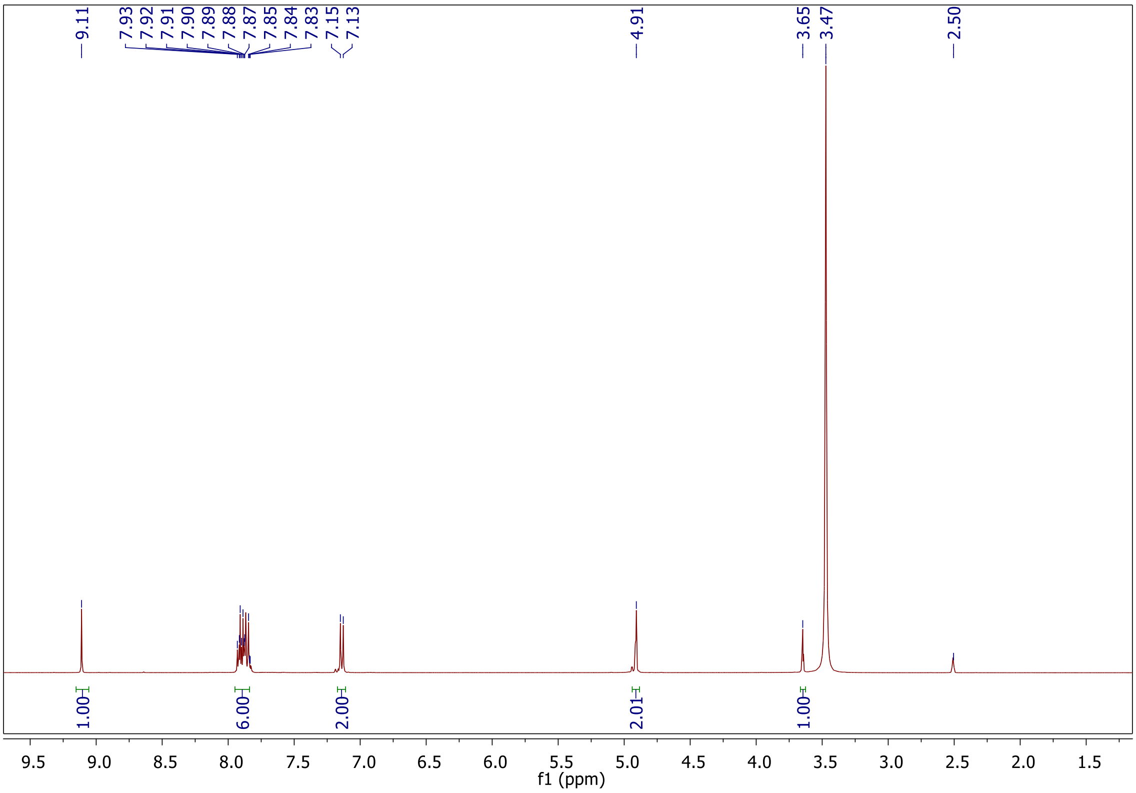

Figure S1: ^{1}H NMR spectrum of compound **1**.

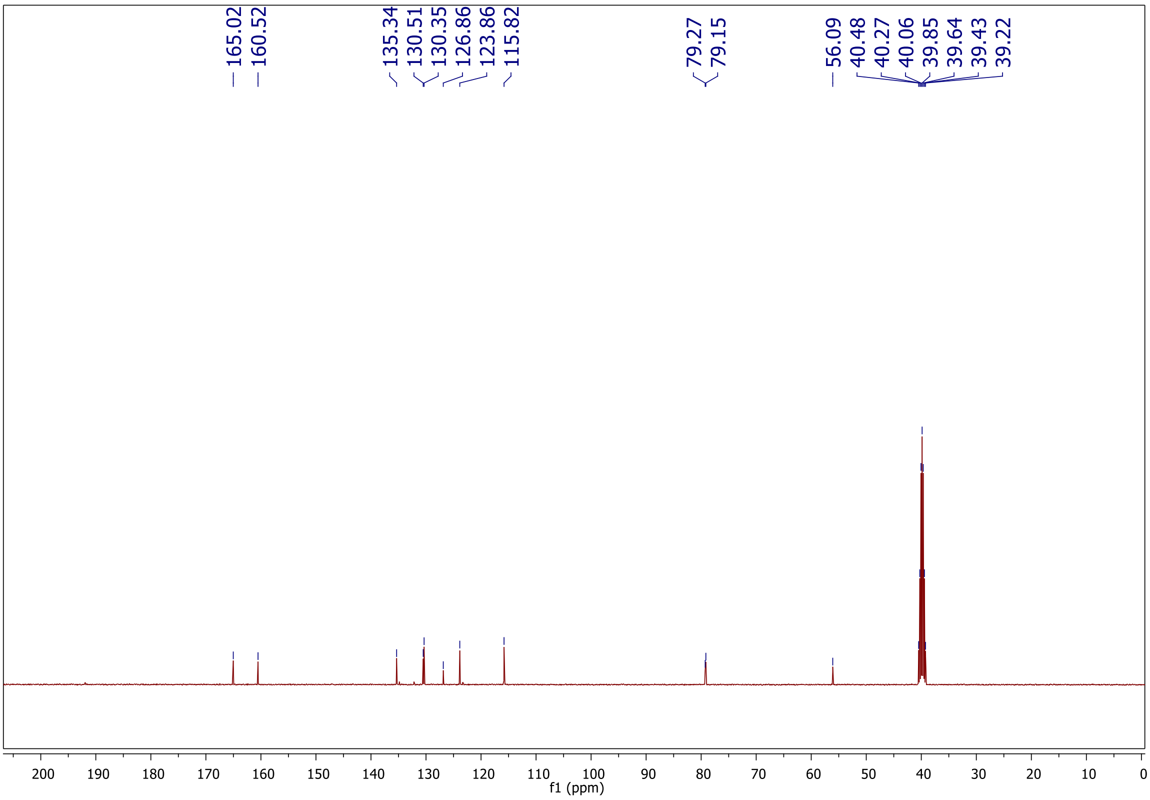

Figure S2: ^{13}C NMR spectrum of compound **4**.

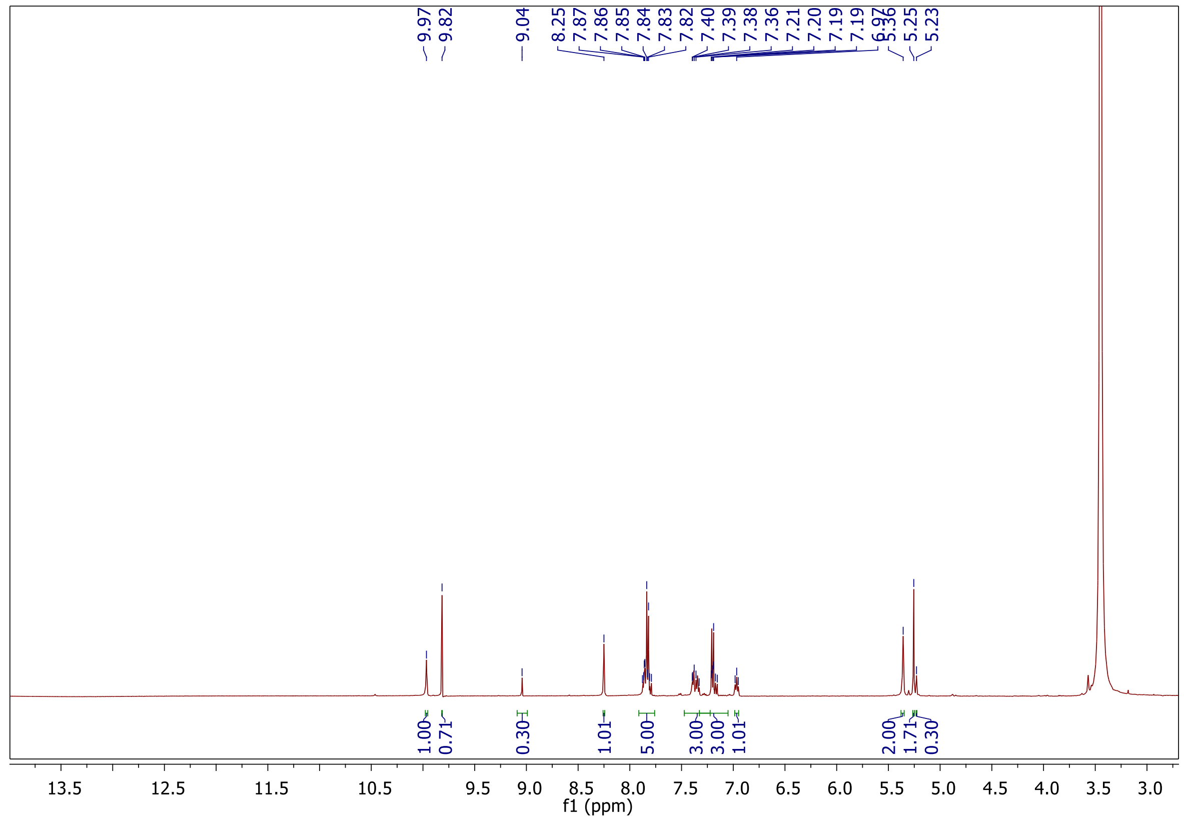

Figure S3: ^{1}H NMR spectrum of compound **6b**.

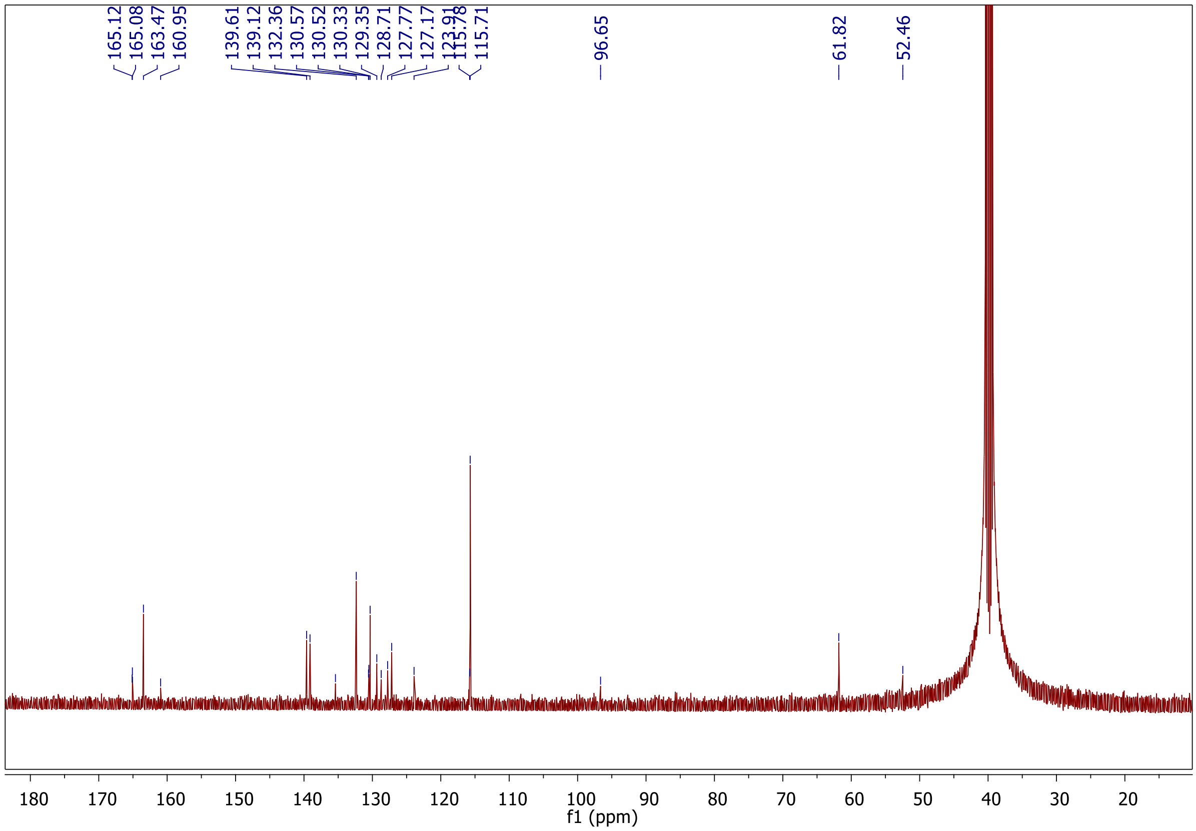

Figure S4: ^{13}C NMR spectrum of compound **6b**.

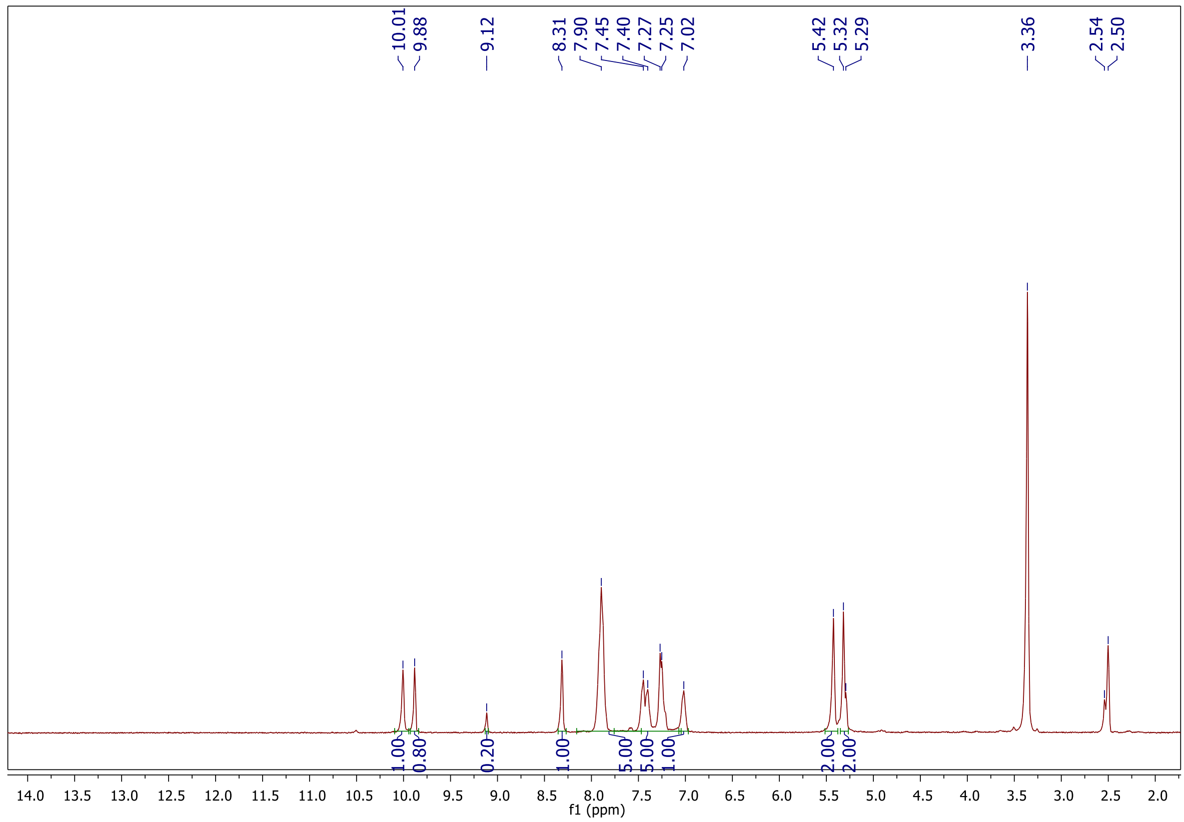

Figure S5: ^{1}H NMR spectrum of compound **6c**.

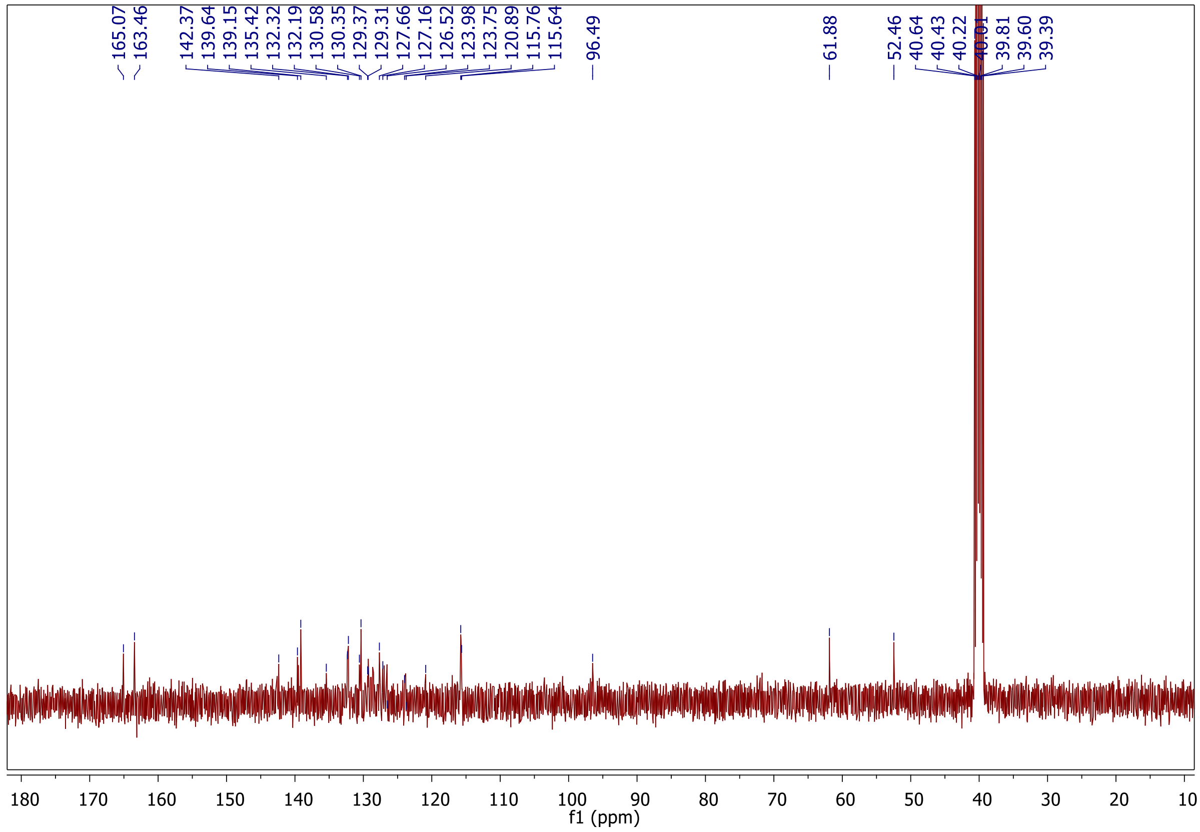

Figure S6: ^{13}C NMR spectrum of compound **6c**.

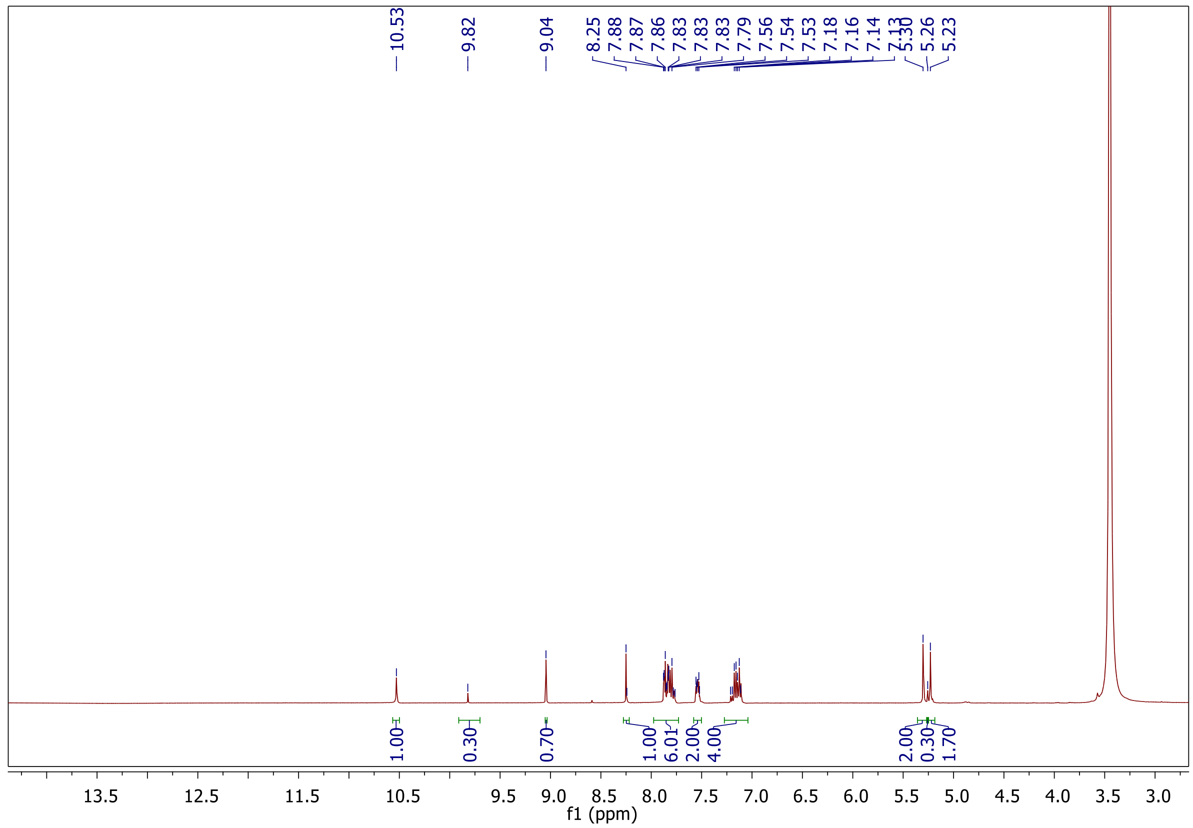

Figure S7: ^{1}H NMR spectrum of compound **6e**.

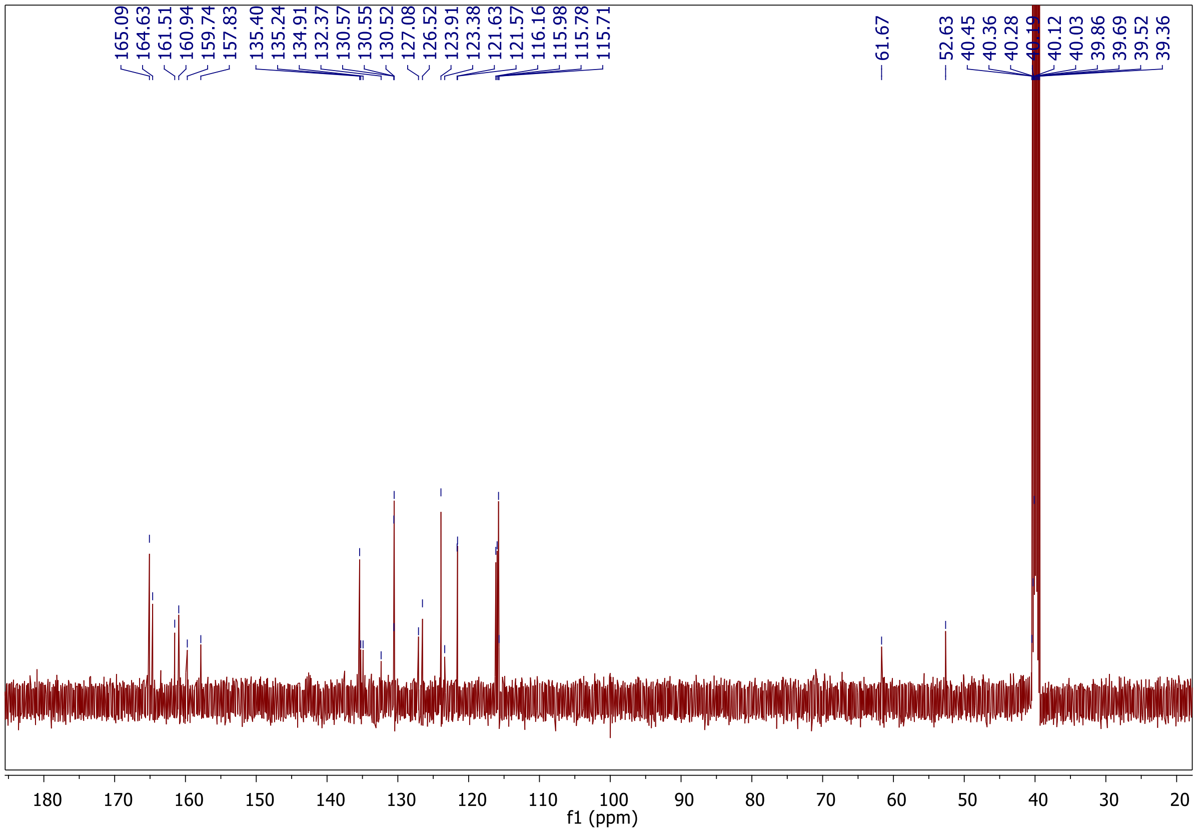

Figure S8: ^{13}C NMR spectrum of compound **6e**.

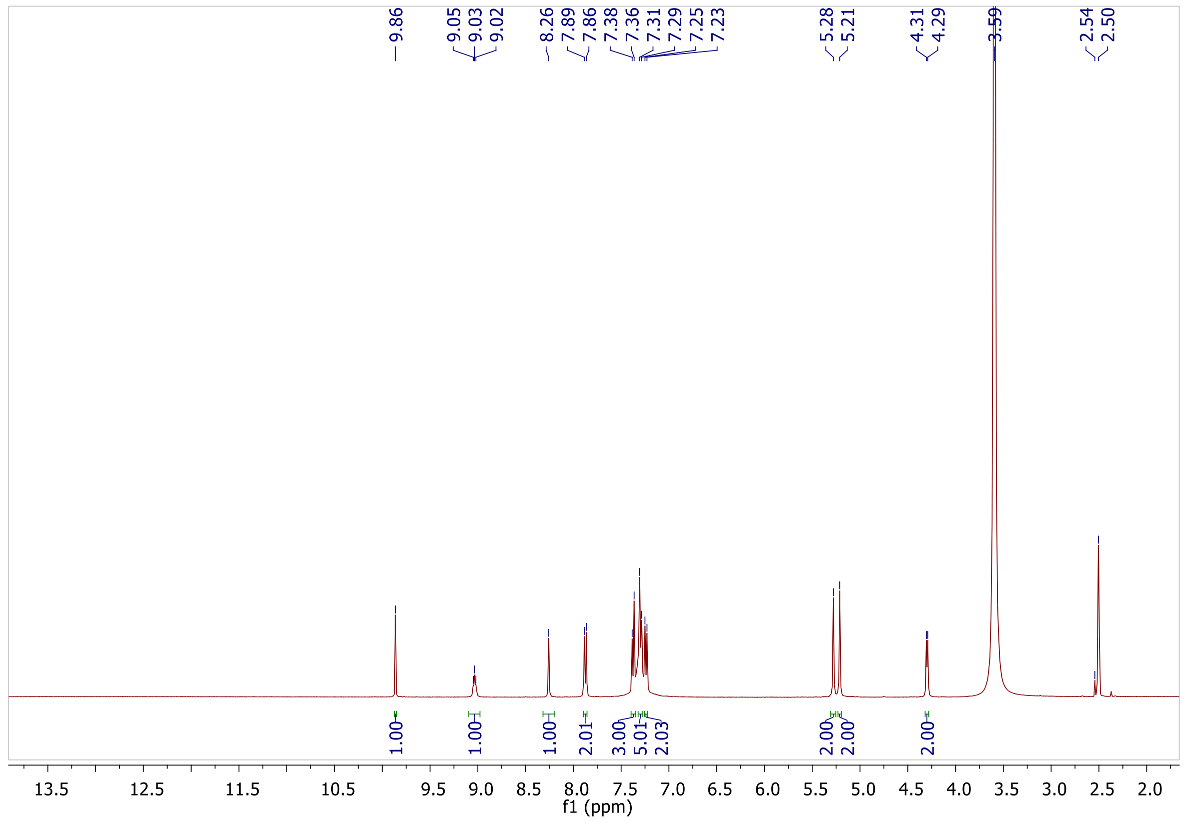

Figure S9: ^{1}H NMR spectrum of compound **6h**.

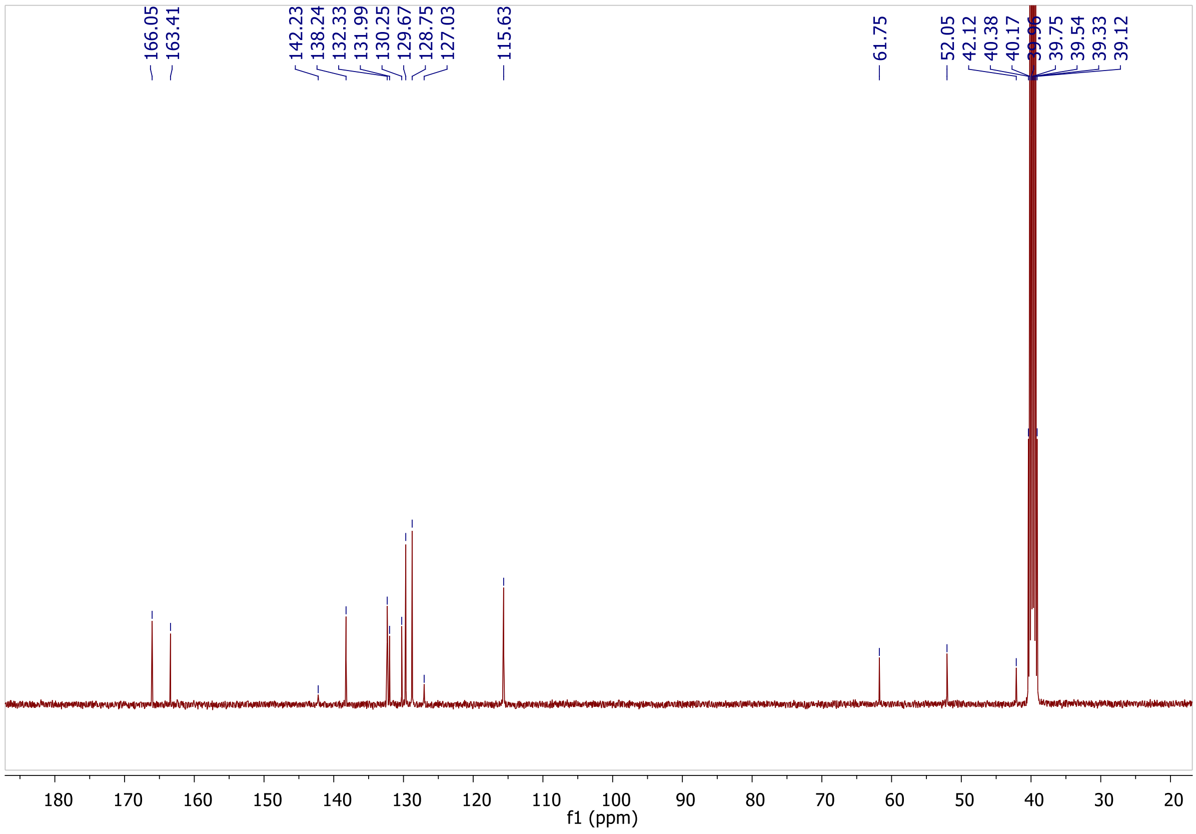

Figure S10: ^{13}C NMR spectrum of compound **6h**.

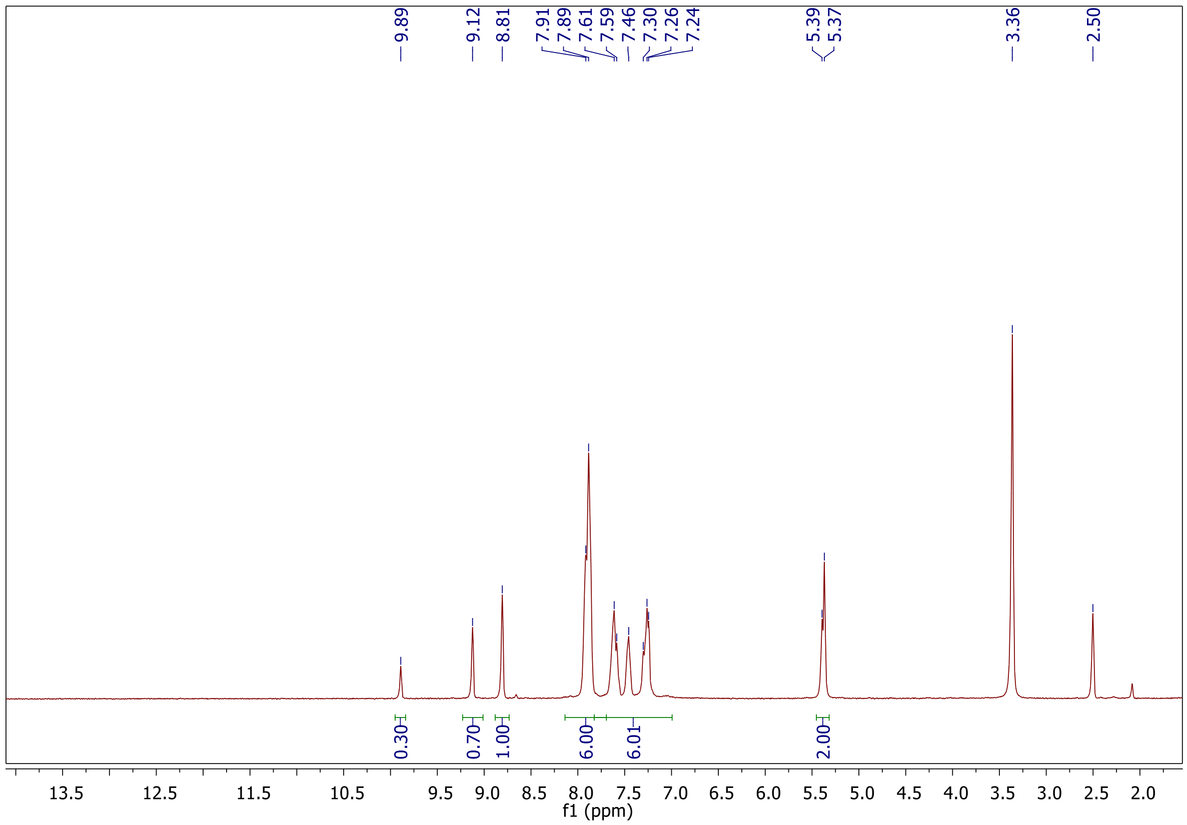

Figure S11: ^{1}H NMR spectrum of compound **8a**.

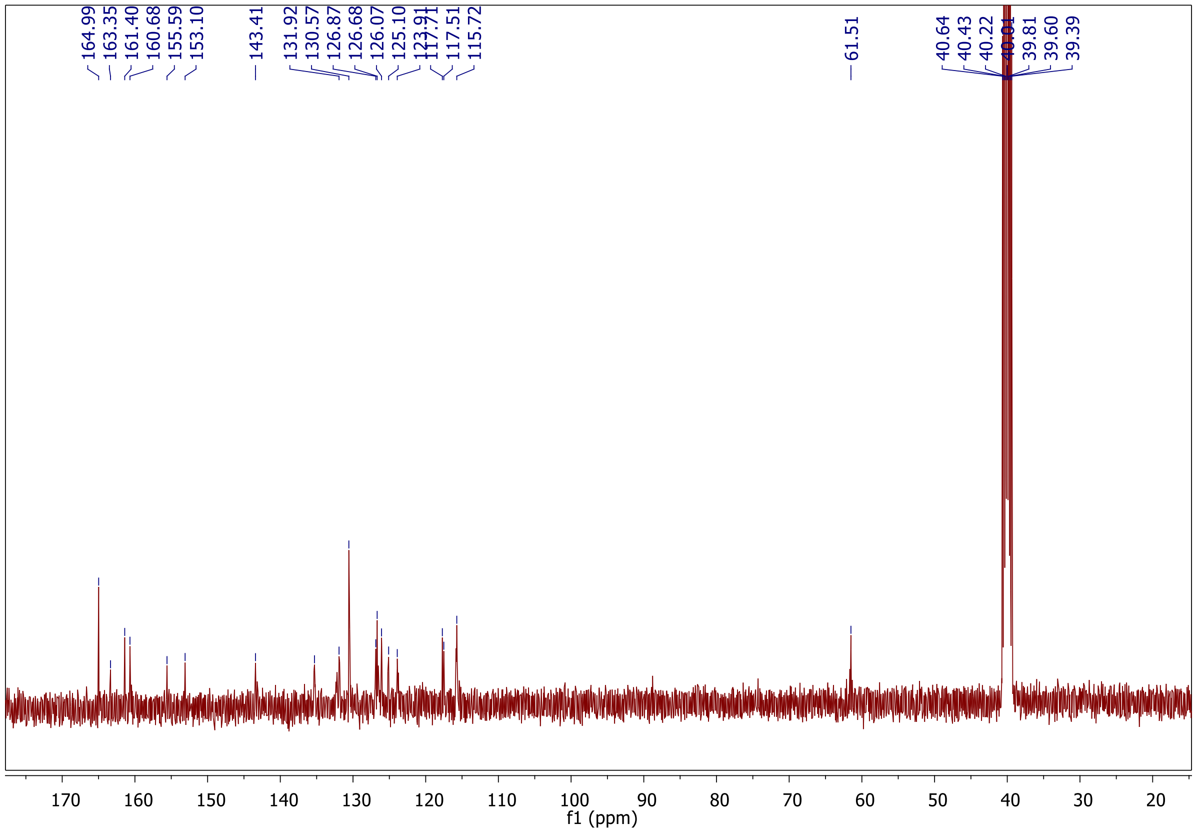

Figure S12: ^{13}C NMR spectrum of compound **8a**.

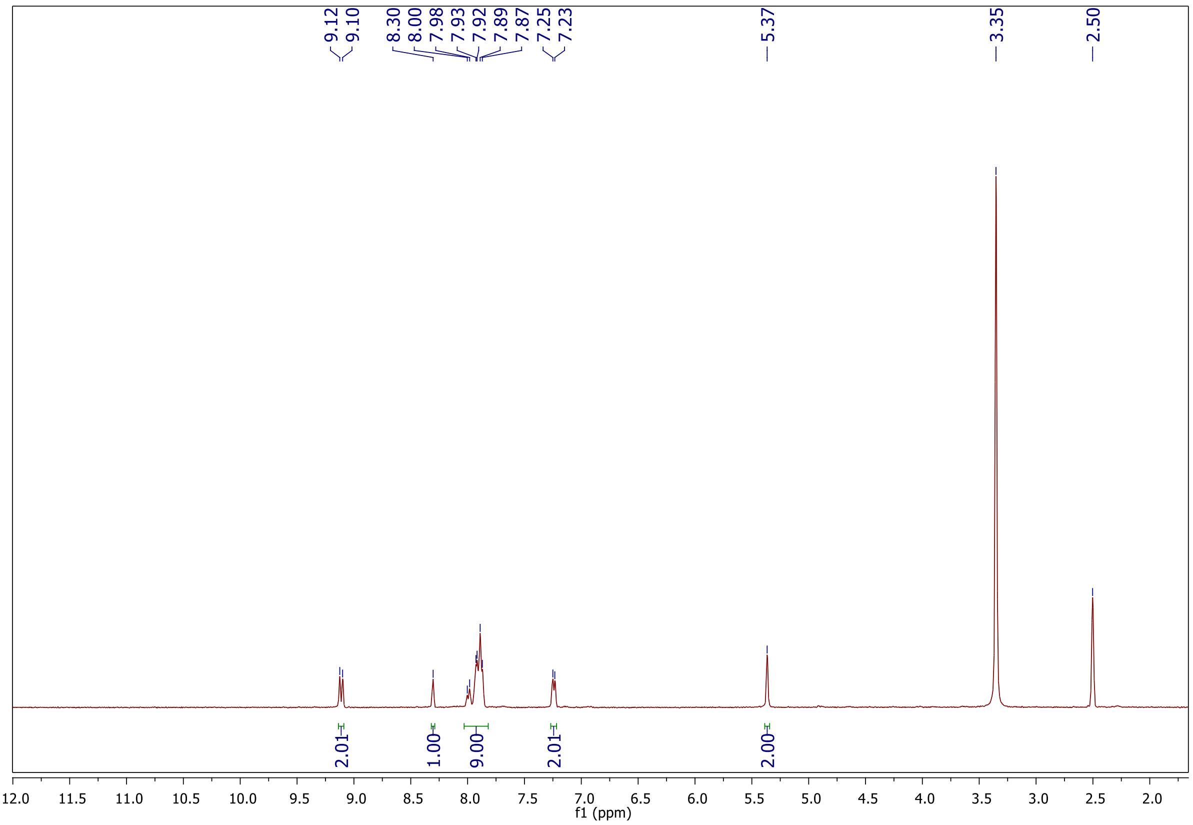

Figure S13: ^{1}H NMR spectrum of compound **8e**.

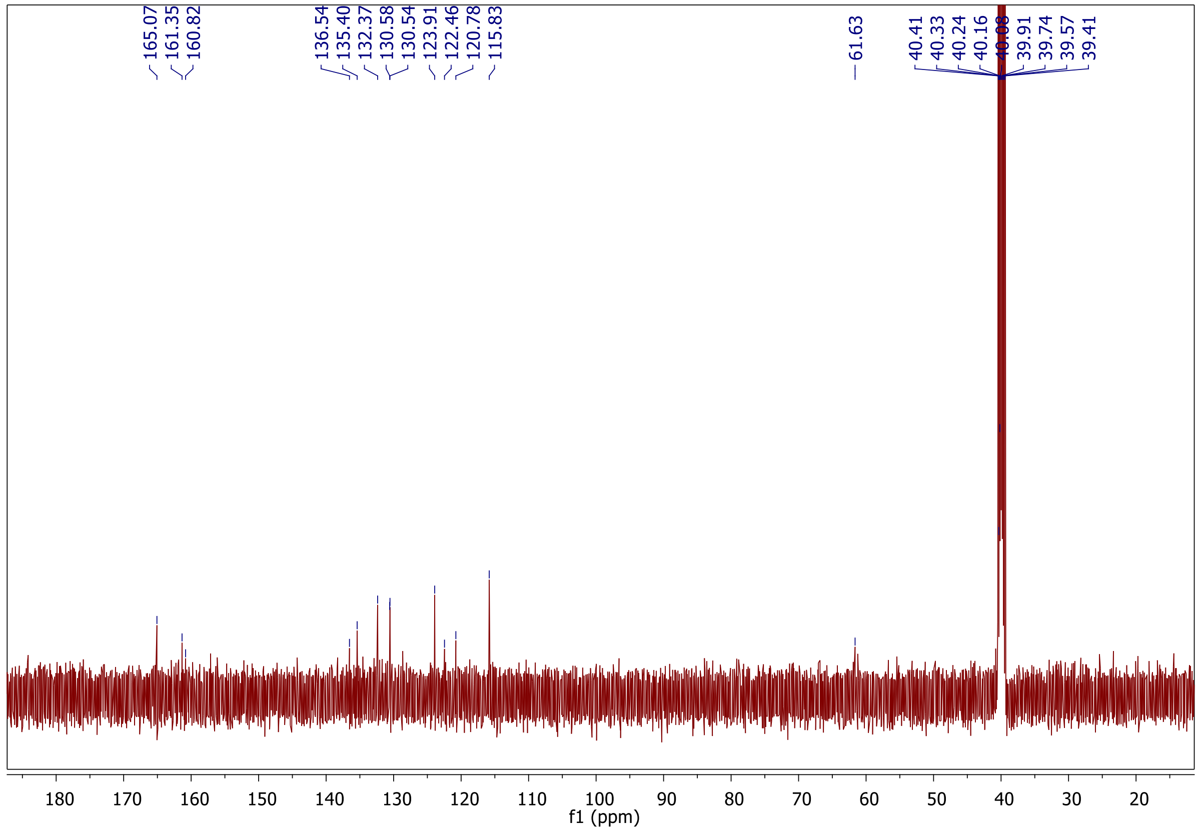

Figure S14: ^{13}C NMR spectrum of compound **8e**.

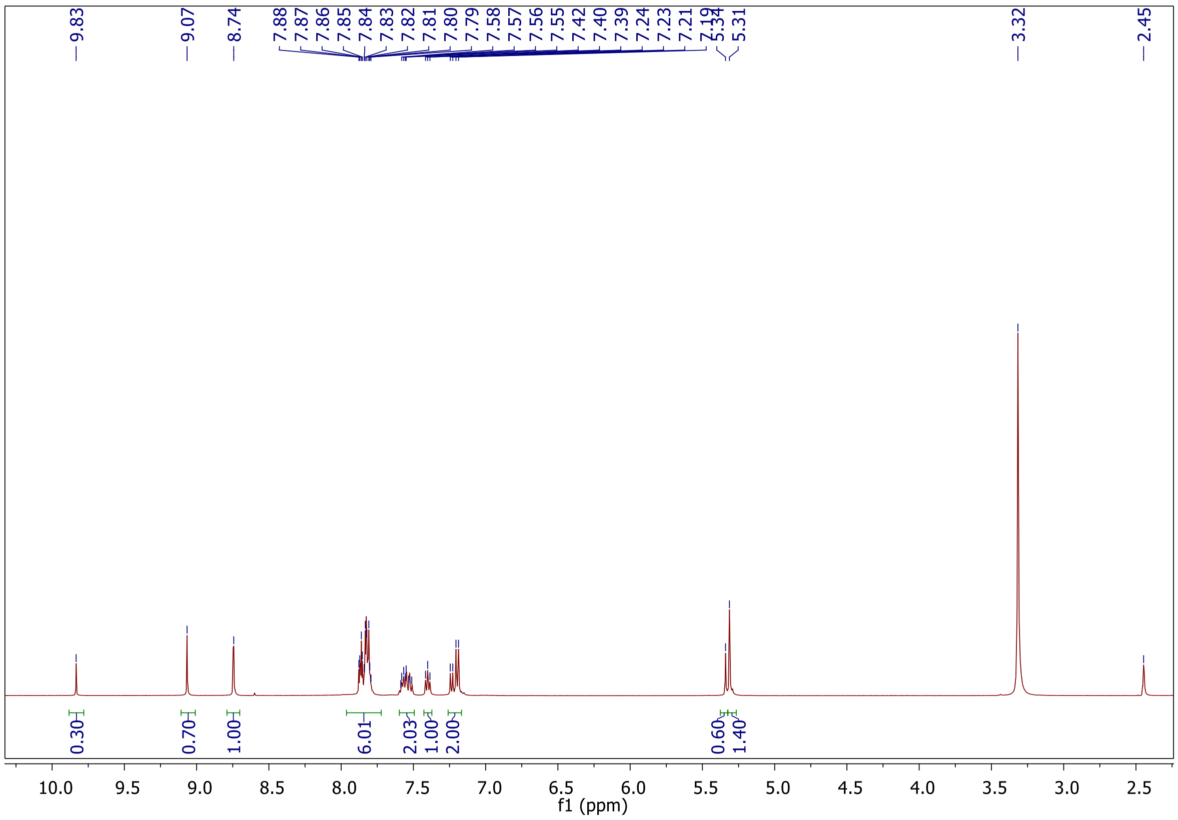

Figure S15: ^{1}H NMR spectrum of compound **8g**.

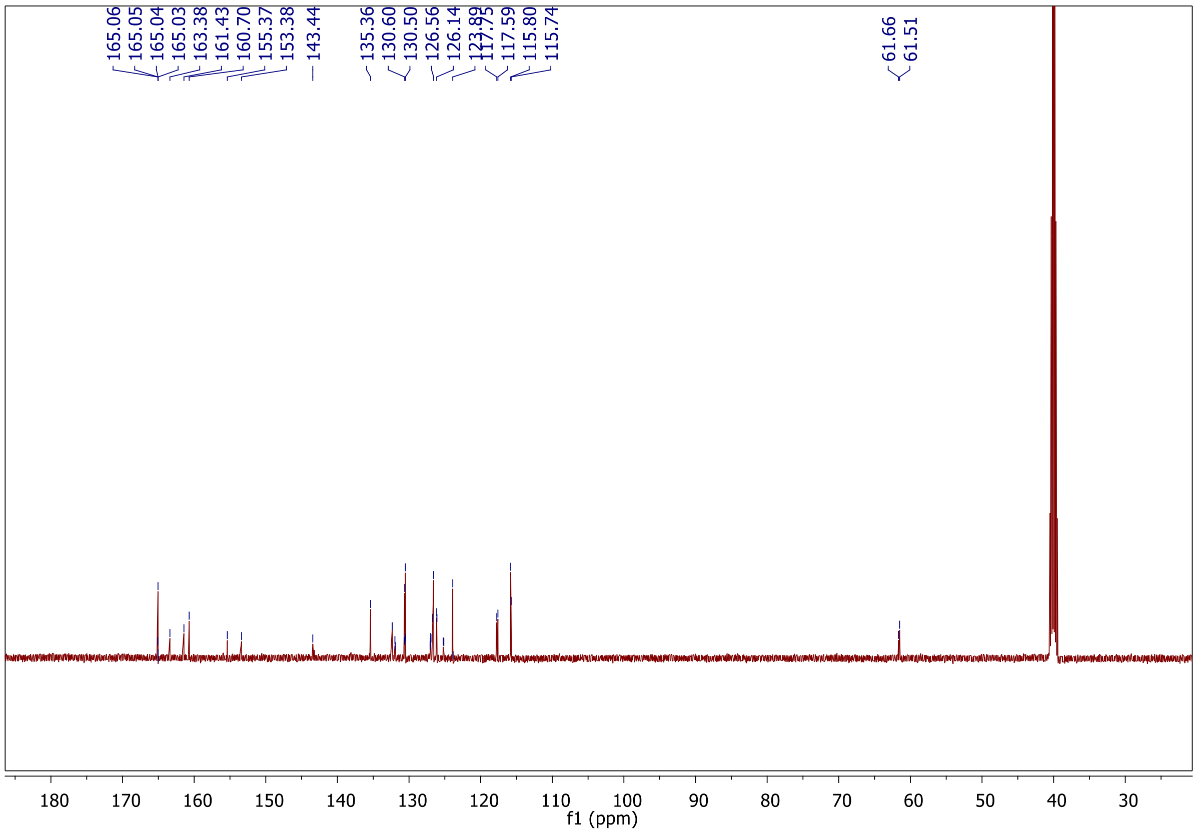

Figure S16: ^{13}C NMR spectrum of compound **8g**.

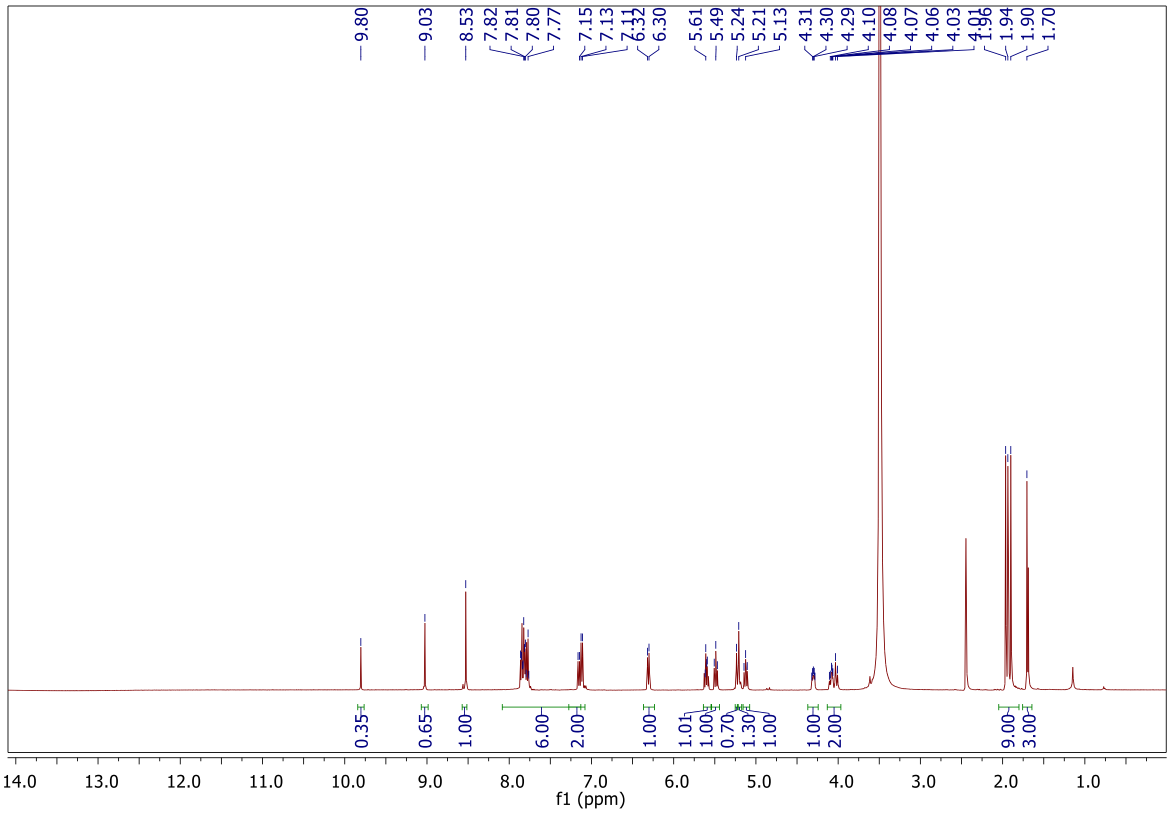

Figure S17: ^{1}H NMR spectrum of compound **10a**.

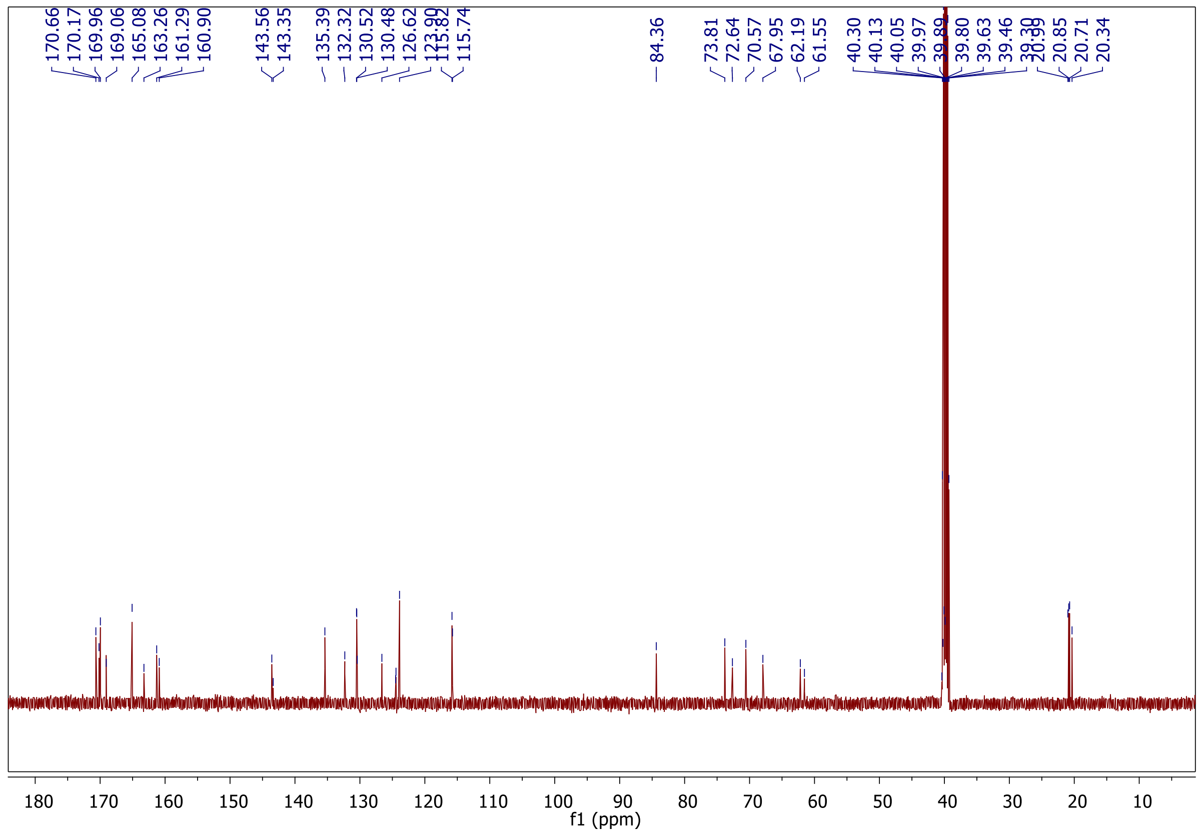

Figure S18: ^{13}C NMR spectrum of compound **10a**.

2. Biological assays

1 M^{pro} protease inhibition assay

The COV2-SARS-CoV-2 protease enzyme assay [1,2] was described in the manufacturing protocol (BPS Bioscience). In summary, it was performed by adding 30 µl of SARS-CoV-2 protease and 10 µl of compound diluted in 1× assay buffer. The solution was then incubated for 30 min at room temperature. The reaction began with the addition of 10 µl of the diluted substrate, which is a M^{pro} self-quenching 14-mer fluorogenic (FRET) peptide with a cleavage site indicated by an arrow (Dabcyl-KTSAVLQ↓SGFRKME-Edans) (BPS Bioscience) using a counter-screen assay for the quenching of inhibitors' fluorescence effect. Then Incubated at room temperature overnight and the plate was sealed with the plate sealer. The fluorescence intensity was measured in a microtiter plate-reading fluorimeter capable of excitation at a wavelength of 360 nm and detection of emission at a wavelength of 460 nm. The fluorescence intensity can also be measured kinetically. The "Blank" value is subtracted from all other values. The experiment was carried out three times to acquire the IC_{50} value, and the error from the global fit with varied hill slope.

2 SARS-CoV-2 Antiviral assay

To assess the effect of different target chemical treatments on SARS-CoV-2 viral load (SARS-CoV-2 isolate EGY/WAT-2 VACCERA), a Real-Time PCR test was used to detect SARS-CoV-2 viral RNA [3,4]. Total RNA was extracted according to the manufacturer's instructions using the genesig® Coronavirus SARS-CoV-2 Real-Time PCR Assay kit (Primer design TM Ltd, Southampton, United Kingdom). This kit includes a master mix, primers, and probe for reverse transcription of extracted RNA as well as Real-Time PCR for SARS-CoV-2 detection. The assay was carried out using the Rotor-Gene Q instrument (Qiagen, Germany) under the following amplification conditions: 10 minutes of reverse transcription at 55 °C, 2 minutes of initial activation at 95 °C, 45 cycles of 10 seconds denaturation at 95 °C and 60 seconds annealing and extension at 60 °C, and 45 cycles of annealing and extension at 60 °C.

References

1. Minvielle, M.J.; Bunders, C.A.; Melander, C. Indole–Triazole Conjugates Are Selective Inhibitors and Inducers of Bacterial Biofilms. *Medchemcomm* **2013**, *4*, 916, doi:10.1039/c3md00064h.
2. Wang, G.; Peng, Z.; Wang, J.; Li, X.; Li, J. Synthesis, in Vitro Evaluation and Molecular Docking Studies of Novel Triazine-Triazole Derivatives as Potential α-Glucosidase Inhibitors. *Eur. J. Med. Chem.* **2017**, *125*, 423–429, doi:10.1016/j.ejmech.2016.09.067.
3. Niedzwiecki In Vitro Modulation of MMP-2 and MMP-9 in Human Cervical and Ovarian Cancer Cell Lines by Cytokines, Inducers and Inhibitors. *Oncol. Rep.* **2010**, *23*, doi:10.3892/or_00000675.
4. Niedzwiecki Patterns of MMP-2 and MMP-9 Expression in Human Cancer Cell Lines. *Oncol. Rep.* **2009**, *21*, doi:10.3892/or_00000358.
